# Infection fatality rate of COVID-19 in community-dwelling elderly populations

**DOI:** 10.1007/s10654-022-00853-w

**Published:** 2022-03-20

**Authors:** Cathrine Axfors, John P. A. Ioannidis

**Affiliations:** 1 Meta-Research Innovation Center at Stanford (METRICS), Stanford University, Stanford, CA USA; 2 Departments of Medicine, of Epidemiology and Population Health, of Biomedical Data Science, and of Statistics, Stanford University, Stanford, CA USA; 3 Stanford Prevention Research Center, Medical School Office Building, Room X306, 1265 Welch Road, Stanford, CA 94305 USA

**Keywords:** COVID-19, Infection fatality rate, Elderly

## Abstract

This mixed design synthesis aimed to estimate the infection fatality rate (IFR) of Coronavirus Disease 2019 (COVID-19) in community-dwelling elderly populations and other age groups from seroprevalence studies. Protocol: https://osf.io/47cgb. Eligible were seroprevalence studies done in 2020 and identified by any of four existing systematic reviews; with ≥ 500 participants aged ≥ 70 years; presenting seroprevalence in elderly people; aimed to generate samples reflecting the general population; and whose location had available data on cumulative COVID-19 deaths in elderly (primary cutoff ≥ 70 years; ≥ 65 or ≥ 60 also eligible). We extracted the most fully adjusted (if unavailable, unadjusted) seroprevalence estimates; age- and residence-stratified cumulative COVID-19 deaths (until 1 week after the seroprevalence sampling midpoint) from official reports; and population statistics, to calculate IFRs adjusted for test performance. Sample size-weighted IFRs were estimated for countries with multiple estimates. Thirteen seroprevalence surveys representing 11 high-income countries were included in the main analysis. Median IFR in community-dwelling elderly and elderly overall was 2.9% (range 1.8–9.7%) and 4.5% (range 2.5–16.7%) without accounting for seroreversion (2.2% and 4.0%, respectively, accounting for 5% monthly seroreversion). Multiple sensitivity analyses yielded similar results. IFR was higher with larger proportions of people > 85 years. The IFR of COVID-19 in community-dwelling elderly is lower than previously reported.

**Supplementary Information:**

The online version contains supplementary material available at 10.1007/s10654-022-00853-w.

## Introduction

Coronavirus Disease 2019 (COVID-19) affects the elderly [1], and nursing homes residents are particularly vulnerable [2]. Numerous seroprevalence studies have been conducted in various populations, locations, and settings. These data have been used and synthesized in several published efforts to obtain infection fatality rate (IFR, proportion of deceased among those infected) estimates [3–6]. All analyses identify very strong risk-gradient based on age, although absolute risk values still have substantial uncertainty. Importantly, the vast majority of seroprevalence studies include very few elderly people [7]. Extrapolating seroprevalence from younger to older age groups is tenuous. Elderly people may genuinely have different seroprevalence. Ideally, elderly should be more protected from exposure/infection, although probably the ability to protect the elderly has varied substantially across countries [8]. Moreover, besides age, comorbidities and lower functional status markedly affects COVID-19 death risk [9, 10]. Particularly long-term care residents accounted for 30–70% of COVID-19 deaths in high-income countries in the first wave [2], despite comprising < 1% of the population. IFR in nursing home residents may be as high as 25% [11]. Not separating persons living in institutions from the community-dwelling may provide an average that is too low for the former and too high for the latter. Moreover, ascertainment and reporting of COVID-19 cases and deaths in long-term care facilities show considerable variation across countries [2], with potentially heavy bearing on overall mortality, while community-dwelling elderly data may be less unreliable (especially in high-income countries). Finally, seroprevalence estimates reflect typically community-dwelling populations (enrollment of people living in institutions is scarce/absent in serosurveys).

Here we estimated the COVID-19 IFR in community-dwelling populations at all locations where seroprevalence studies with many elderly individuals have been conducted.

## Methods

### Information sources

We identified seroprevalence studies (peer-reviewed publications, official reports, or preprints) in four existing systematic reviews [3, 7, 12, 13] as for a previous project [14], using the most recent updates of these reviews and their respective databases as of November 23, 2021. All systematic reviews may miss some studies, despite their systematic efforts. Here, the risk is minimized by using several existing systematic reviews of seroprevalence studies, each of them very meticulous. The protocol of this study was registered at the Open Science Framework (https://osf.io/47cgb) after piloting data availability in December 2020 but before extracting full data, communicating with local authorities and study authors for additional data and performing any calculations. Protocol amendments and their justification appear in Online Appendix Table 1.

### Eligibility criteria

In the original protocol, we aimed to include studies on SARS-CoV-2 seroprevalence that had sampled at least 1000 participants aged ≥ 70 years in the location and/or setting of interest, provided an estimate of seroprevalence for elderly people, explicitly aimed to generate samples reflecting the general population, and were conducted at a location for which there is official data available on the proportion of cumulative COVID-19 deaths among elderly (with a cutoff placed between 60 and 70 years; e.g., eligible cutoffs were ≥ 70, ≥ 65, or ≥ 60, but not ≥ 75 or ≥ 55). Following comments from peer-reviewers, we also included national-level general population studies without high risk of bias and with at least 500 participants aged ≥ 70 years (since studies with 500–1000 may still yield reasonably precise estimates of seroprevalence in the elderly) and we excluded samples of patient cohorts, insurance applicants, blood donors, and workers (that were intended for inclusion in the original protocol). These convenience samples with ≥ 1000 participants aged ≥ 70 years are now considered only in a sensitivity analysis. USA studies were excluded if they did not adjust seroprevalence for race or ethnicity, since these socio-economically related factors may associate strongly with both study participation [15, 16] and COVID-19 burden [17–19]. Following comments from peer-reviewers, we have added another exclusion criterion: crude seroprevalence being less than 1-test specificity and/or the 95% confidence interval of the seroprevalence going to 0% (since the seroprevalence estimate would be extremely uncertain). Following comments from peer-reviewers, we also considered only studies from high-income countries in the primary analysis, since there can be substantial uncertainty about the number of deaths in other countries. We focused on studies sampling participants in 2020, since IFRs in 2021 may be further affected by wide implementation of vaccinations that may substantially decrease fatality risk and by other changes (new variants and better treatment). We applied risk of bias assessments reported by SeroTracker (based on the Joanna Briggs Institute Critical Appraisal Tool for Prevalence Studies) [20]. Two authors reviewed records for eligibility. Discrepancies were solved by discussion.

### Data extraction

CA extracted each data point and JPAI independently verified the extracted data. Discrepancies were solved through discussion. For each location, we identified the age distribution of cumulative COVID-19 deaths and chose as primary age cutoff the one closest to 70, while placed between 60 and 70 years (e.g., ≥ 70, ≥ 65, or ≥ 60).

Similar to a previous project [3], we extracted from eligible studies information on location, recruitment and sampling strategy, dates of sample collection, sample size (overall and elderly group), and types of antibody measured (immunoglobulin G (IgG), IgM and IgA). We also extracted, for the elderly stratum, the estimated unadjusted seroprevalence, the most fully adjusted seroprevalence, and the factors considered for adjustment. Antibody titers may decline over time. E.g. a modelling study estimated 3–4 months average time to seroreversion [21]. A repeated measurements study [22] suggests even 50% seroreversion within a month for asymptomatic/oligosymptomatic patients, although this may be an over-estimate due to initially false-positive antibody results. To address seroreversion, if there were multiple different time points of seroprevalence assessment, we selected the one with the highest seroprevalence estimate. If seroprevalence data were unavailable as defined by the primary cutoff, but with another eligible cutoff (e.g., ≥ 70, ≥ 65, or ≥ 60), we extracted data for that cut-off.

Population size (overall, and elderly) and numbers of long-term care residents for the location were obtained from multiple sources (Online Appendix Table 2).

Cumulative COVID-19 deaths overall and in the elderly stratum (using the primary age cutoff) for the relevant location were extracted from official reports. Total numbers, i.e., confirmed and probable, was preferred whenever available. We extracted the accumulated deaths until 1 week after the midpoint of the seroprevalence study period (or closest date with available data) to account for different delays in developing antibodies versus dying from infection [23, 24]. If the seroprevalence study claimed strong arguments to use another time point or approach, while reporting official statistics on number of COVID-19 deaths overall and in the elderly, we extracted that number instead.

The proportion of cumulative COVID-19 deaths that occurred among long-term care residents for the relevant location and date was extracted from official sources or the International Long Term Care Policy Network (ILTCPN) report closest in time [2, 25]. We defined community-dwelling individuals by excluding persons living in institutions. Types of institutions used for elderly in various countries differed in nature and in the frailty of residents. For each location, we extracted available definitions of institutions. We preferred numbers recorded per residence status, i.e., including COVID-19 deaths among nursing home residents occurring in hospital. If the latter were unavailable, we calculated total number of deaths in residents with a correction (by multiplying with the median of available ratios of deaths in nursing homes to deaths of nursing home residents in the ILTCPN 10/14/2020 report [2] for countries in the same continent). We considered 95%, 98%, and 99% of residents’ deaths to have occurred in people ≥ 70 years, ≥ 65 years and ≥ 60 years, respectively [26]. For other imputations, see the online protocol.

### Missing data

We communicated with the authors of the seroprevalence study and with officers responsible for compiling the relevant official reports to obtain missing information or when information was available but not for preferred age cut-offs. Email requests were sent, with two reminders to non-responders.

### Calculated data variables

#### Infected and deceased community-dwelling elderly

The number of infected people among the community dwelling elderly for the preferred date (1 week after the midpoint of the seroprevalence study period) was estimated by multiplying the adjusted estimate of seroprevalence and the population size in community-dwelling elderly. We preferred the most adjusted seroprevalence estimates. Following a suggestion from peer-reviewers, whenever no adjustment was made for test performance, we adjusted the estimates for test performance using the Gladen–Rogan formula [27] and did not correct for types of unmeasured antibodies (IgG/IgM/IgA) [3, 28]. Moreover, we applied a non-prespecified correction for studies that excluded persons with diagnosed COVID-19 from sampling, primarily by using study authors’ corrections, secondarily by adding the number of identified COVID-19 cases in community-dwelling elderly for the location up to the seroprevalence study midpoint.

The total number of fatalities in community-dwelling elderly was obtained by total number of fatalities in elderly minus those accounted for by long-term care residents in the elderly stratum. If the elderly proportion or residents’ share of COVID-19 deaths were only available for another date than the preferred one, we assumed the proportions were stable between time points.

#### IFR estimation

We added a non-prespecified calculation of 95% confidence intervals (CIs) of IFRs based on extracted or calculated 95% CIs from seroprevalence estimates (Online Appendix Table 1). No further factors were introduced in the calculation beyond Gladen–Rogan corrections where no adjustment had been done for test performance. CI estimates should be seen with caution since they depend on adequacy of seroprevalence adjustments, and do not consider other types of uncertainty (e.g., regarding mortality statistics).

### Synthesis of data

Statistical analyses used R version 4.0.2 [29]. Similar to a previous overview of IFR-estimating studies [3], we estimated the sample size-weighted IFR of community-dwelling elderly for each country and then estimated the median and range of IFRs across countries. As expected, there was extreme heterogeneity among IFR estimates (I^2^ = 99.1%), thus weighted meta-analysis averages are not meaningful [30, 31].

We explored a seroreversion correction of the IFR by X^m^-fold, where m is the number of months from the peak of the first epidemic wave in the specific location and X is 0.99, 0.95, and 0.90 corresponding to 1%, 5%, and 10% relative monthly rate of seroreversion [21, 22, 32]. We also added a non-prespecified sensitivity analysis to explore the percentage increase in the cumulative number of deaths and IFR, if the cutoff was put 2 weeks (rather than 1 week) after the study midpoint.

We expected IFR would be higher in locations with a higher share of people ≥ 85 years old among the analyzed elderly stratum. Estimates of log_10_IFR were plotted against the proportion of people ≥ 85 years old among the elderly (for population pyramid sources see Online Appendix Table 2).

### Ethics approval

Not applicable to this study.

### Patient and public involvement

There was no involvement of patients nor the public in this research.

## Results

### Seroprevalence studies

By November 23, 2021, 3138 SARS-CoV-2 seroprevalence reports were available in the four systematic reviews. Screening and exclusions are shown in Online Appendix Figure 1 and Online Appendix Table 3. Twelve seroprevalence studies were included, one of which contained two separate surveys; another 13 studies with convenience samples were considered in sensitivity analysis [33, 34, 35, 36, 37, 38, 39, 40, 41, 42, 43, 44, 45, 46, 47, 48–56].

Table 1 shows for each study the sampling period, sample size tested and positive, age cut-offs for the elderly group, antibody type(s), seroprevalence estimates, types of adjustments, number of deaths, and IFR estimates. The 13 seroprevalence surveys in the main analysis (Table 1) represented 11 countries (Americas n = 2, Europe n = 11). Three studies excluded upfront persons with previously diagnosed COVID-19 from participating in their sample [53, 57, 58]. Mid-sampling points ranged from May 2020 to November 2020. Sampling had a median length of 5.4 weeks (range 13 days to 5 months). The median number of elderly individuals tested was 1473 (range 788–21,953). Median seroprevalence was 3.2% (range 0.47–14.9%). Adjusted seroprevalence estimates were available for 12/13 surveys.

**Table 1:** Included seroprevalence studies with estimates of seroprevalence in the elderly, COVID-19 deaths in the elderly and community-dwelling elderly, and corrected infection fatality rate

Location (first author)	Recruitment strategy	Sampling period	Number tested; number positive (n)	Age cutoff for mortality; age cutoff for seroprevalence (years)	Antibody type(s)	Adjusted seroprevalence; crude seroprevalence (%)	Adjustments	Deaths in community-dwelling elderly [all elderly] (n)	Population, community-dwelling elderly [all elderly] (n)	IFR community-dwelling elderly [all elderly] (%)
*Main analysis*										
Andorra (Royo-Cebrecos)*	General	May 4 to May 28	4339; 582	70; 70	IgG/IgM	NA; 14.92	None	22 [45]	7364 [7631]	2.02 [3.95]
Ontario, Canada (Public Health Ontario, COVID-19 Immunity Task Force)	General	June 5 to June 30	1236; 25	70; 70	IgG	1.92; 2.02	Population weighting and test characteristics	525 [2298]	1,392,596 [1513920]	1.96 [7.89]
Denmark (Espenhain)	General	Median date Sept 19, sampling period approx. 13 weeks	1473; NA	70; 65	IgG/IgM/IgA	1.5; NA	Test sensitivity and specificity	350 [565]	798,797 [836716]	2.92 [4.5]
France (Warszawski, INSERM)*	General	Median date Nov 24, interquartile range Nov 18 to Dec 4	14,531; 611	65; 65	IgG	5.05; 5.27	Sociodemographics, income, quality of contact information, population density, proportion of people below poverty line, age, gender, "departement", educational level, region	26,958 [49488]	12,902,526 [13440786]	4.14 [7.29]
Ile-de-France, France (Carrat)	General	May 4 to June 23 (90% of tests were performed May 4 to May 24)	1394; 52	70; 70	IgG	4.73; 3.73	Age, sex, socio-professional category, test sensitivity and specificity	4297 [7712]	1,279,740 [1339192]	7.1 [12.18]
Nouvelle-Aquitaine, France (Carrat)	General	May 4 to June 23 (90% of tests were performed May 4 to May 24)	1765; 29	65; 65	IgG	1.7; 1.64	Age, sex, socio-professional category, test sensitivity and specificity	303 [409]	1,406,958 [1465885]	1.27 [1.65]
Hungary (Merkely)*	General	May 1 to May 16	1454; 9	70; 70	IgG	1.12; 0.93	"several area-, dwelling unit-, and individual-level auxiliary information", region, sex, age	248 [348]	1,198,425 [1249016]	1.85 [2.48]
Iceland (Gudbjartsson)	General	May 5 to June 12 (healthcare sample)	NA; NA	70; 70	IgG/IgM/IgA	0.47; NA	Region, sex, age	5 [7]	32,782 [34865]	3.12 [4.23]
Italy (ISTAT)	General	May 25 to July 15	NA; NA	70; 70	IgG	2.5; NA	Region, municipal type, gender, age group, employment status, municipal prevalence, percentage difference in municipal mortality rates compared to the same period of the previous year	19,341 [29722]	10,136,405 [10400756]	7.63 [11.43]
Netherlands (Vos)	General	June 9 to August 24; 90% enrolled by June 22	788; NA	70; 70	IgG	5; NA	Sex, age, ethnic background, degree of urbanization, test characteristics	2664 [5402]	2,346,712 [2451000]	2.27 [4.41]
Spain (ISCII)*	General	November 16 to November 29	7526; NA	70; 70	IgG	7.88; NA	Province, sex, age, income	23,335 [41681]	6,512,456 [6823002]	4.55 [7.75]
England (Ward)	General	June 20 to July 13	21,953; 801	70; 65	IgG	3.25; 3.65	Test performance, age, sex, region, ethnicity, deprivation	22,644 [41023]	7,204,057 [7556976]	9.68 [16.72]
USA (Kalish)	General	April 1 to August 2020 (> 88% between May 10 and July 31)	1273; 46	65; 70	IgG/IgM/IgA	3.5; 3.61	Region, age, sex, race, ethnicity, urban/rural, children, education, homeowner, employment, health insurance, health-related questions, test performance	46,571 [103862]	52,441,191 [54058263]	2.27 [4.42]
*Sensitivity analysis (Non-peer-reviewed pre-specified selection criteria)*										
Belgium (Herzog)	Residual blood samples	Mar 30 to Apr 5	1210; 29	70; 70	IgG	1.92; 2.4	Age, sex, province, test sensitivity and specificity	1057 [3317]	1,453,077 [1581078]	3.79 [10.92]
Canada (Saeed, Canadian Blood Services)	Blood donors	May 9 to July 21 (median date June 13)	9845; 74	70; 65	IgG	0.77; 0.75	Residential postal code, age, sex, sensitivity and specificity of the assay	890 [7477]	3,577,421 [3963155]	3.23 [24.5]
Alberta, Canada (Charlton)*	Residual blood samples	December 7 to December 10	2820; 29	70; 70	IgG	1.54; 1.54	None (crude)	213 [823]	289,046 [326530]	4.8 [16.41]
Denmark (Pedersen)	Blood donors	June 2 to June 19	1201; 22	70; 70	IgG/IgM/IgA	1.4; 1.8	Sensitivity and specificity of the diagnostic assay; population size of recruitment areas (municipalities)	329 [531]	530,764 [555882]	4.43 [6.82]
Dominican Republic (Paulino-Ramirez)*	General, hotspot areas	April to June	2739; 164	60; 60	IgG	NA; 10.53	None	237 [282]	1,156,871 [1158933]	0.19 [0.23]
India (Murhekar)	General	August 19 to September 20	2768; 291	61; 61	IgG	6.2; 10.51	Sampling district, test performance	33,655 [41386]	125,239,515 [125325806]	0.43 [0.53]
Tamil Nadu, India (Malani)	General	October 19 to November 30	1568; NA	70; 70	IgG	25.2; NA	Age, gender, test performance, district	3518 [4326]	4,324,265 [4328822]	0.32 [0.4]
Israel (Reicher)*	General	Median date July 9	6937; NA	70; 70	IgG	2.22; NA	Sex, age, municipal strata and RT-PCR status	163 [327]	652,486 [689587]	1.12 [2.14]
Qatar (Abu-Raddad)*	Residual blood samples	May 12 to July 12 (median day June 28)	1809; 162	70; 70	IgG	13.29; 9.15	Sex, age, nationality	53 [65]	18,166 [18247]	2.18 [2.67]
UK (UK Biobank)	Biobank	May 27 to Aug 14 (however monthly repeated sampling)	3956; NA	65; 70	Missing/Unclear	6.1; NA	Unclear	25,678 [49669]	11,917,570 [12374961]	3.53 [6.58]
England and Wales (Public Health England)	Residual blood samples	May 1 to May 30	1702; NA	70; 70	Missing/Unclear	3.64; NA	Population-weighted adjusted	21,063 [37838]	7,665,426 [8037210]	7.55 [12.94]
Greater Glasgow and Clyde, Scotland (Hughes)	Residual blood samples	March 16 to May 24	2771; NA	70; 65	IgG	5.45; 8.23	Test performance, population-level dynamics, sex, age, care type, week of sample collection	295 [627]	188,673 [195952]	2.87 [5.87]
USA (Anand)*	Hemodialysis	July (> 80% in first 2 weeks)	13,659; 1043	65; 65	IgG/IgM/IgA	8.09; 7.65	Age, sex, geographical region, race and ethnicity	51,128 [111774]	52,441,191 [54058263]	1.2 [2.55]

Dominican Republic (Paulino-Ramirez): Number tested (n = 2739) refers to individuals ≥ 55 years old, while number positive (n = 164) refers to those ≥ 60 years old. France (INSERM): The total number of deaths in elderly is derived from their Tableau 4 (deaths occurring in hospital) and Tableau 2 (deaths in care homes). France (Carrat, Ile-de-France): see Online Appendix Table 2 for our calculation of deaths in elderly and community-dwelling elderly. Iceland (Gudbjartsson): Estimate is based on seroprevalence and PCR testing; persons previously diagnosed with COVID-19 did not enroll in the study. UK (Hughes): COVID-19 death statistics for nursing home residents did not include deaths occurring in hospital, and so was corrected with a factor of 1.225 (the median of the ratio of deaths in nursing home residents / deaths occurring in nursing homes, in the European countries with such data in Comas-Herrera et al. International Long-Term Care Policy Network report, October 14). USA (Kalish): Excluded previously COVID-19-diagnosed persons from participating, why we added cases in community-dwelling elderly up to the study midpoint to the number of infected. USA (Anand; Kalish): Estimates for nursing home deaths in the USA are lower in the US Center for Medicare and Medicaid Services (CMS), but CMS counts do not capture many early deaths in long-term care facilities and include only deaths in federally regulated nursing homes, excluding deaths in assisted-living, resident care, and other care facilities, therefore we used information from the Kaiser Family Foundation (KFF) that considers all long-term care facilities

*NA* Not applicable (missing)

^*^Seroprevalence corrected for test performance with the Gladen–Rogan formula since the original source had not done so

### Mortality and population statistics

COVID-19 deaths and population data among elderly at each location are shown in Table 1 (for sources, see Online Appendix Table 2). The proportion of a location’s total COVID-19 deaths that happened among elderly had a median of 86% (range 70–93%) in high-income countries. The proportion of a location’s total COVID-19 deaths that occurred in long-term care residents had a median of 39% (range 20–67%) in in high-income countries with available data (for Qatar, the number was imputed). One study [55] included only COVID-19 deaths that occurred in nursing homes and was corrected to reflect also the deaths among residents occurring in hospitals. Among the population, the elderly group comprised a median of 14% (range 10–24%) in high-income countries. People residing in facilities were 4.8% (range 2.9–8.8%) in high-income countries.

### Additional data contributed

Additional information was obtained from authors and agencies on four studies for seroprevalence data [50, 56, 59, 60]; four studies for mortality data [49, 50, 34, 43]; two studies for population data [49, 50]; and five excluded studies (clarifying non-eligibility).

### Calculated IFRs

**Fig. 1:**
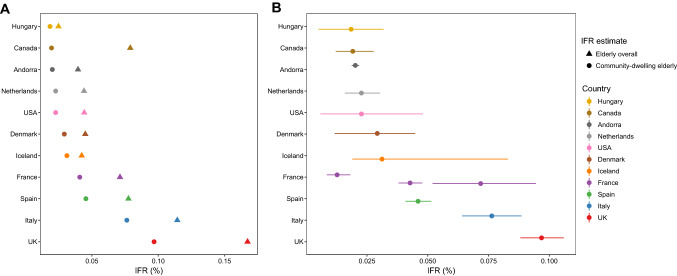
Infection fatality rates (IFRs) in elderly, corrected for unmeasured antibody types. **a** Countries’ IFRs in community-dwelling elderly and elderly overall. **b** IFRs in community-dwelling elderly with 95% confidence intervals based on individual seroprevalence estimates and their uncertainty. If multiple seroprevalence studies were available for the same country, we calculated the sample size-weighted IFR. As per above, the 95% CIs do not take into account other sources of uncertainty than those adjusted by the seroprevalence study authors (except adding an adjustment for test performance as per the Gladen–Rogan formula for those that had not already adjusted for test performance), and should be interpreted as conservative. Primarily, 95% confidence intervals are direct extractions from the seroprevalence studies. For studies that did not report 95% confidence intervals, we complemented with a calculation using the number of sampled and seropositive elderly individuals. For those that provided adjusted estimates for age brackets (e.g., 70–79, 80–89, and 90+), we combined estimates for each study using a fixed effects inverse variance meta-analysis (of arcsine transformed proportions) to obtain 95% CIs. Asymmetry to point estimates may be observed for these cases, since point estimates were calculated by multiplying age bracket seroprevalence by the corresponding population count (which is preferable, since it takes into account population distribution)

For countries with more than one IFR estimate available sample size-weighted average IFRs were calculated. In 11 high-income countries, IFRs in community-dwelling elderly (Fig. 1, Table 1) had a median of 2.9% (range 1.8–9.7%). Figure 1 also shows 95% CIs for IFRs based on 95% CIs for seroprevalence estimates. For 5 studies, 95% CIs were direct extractions from the seroprevalence studies themselves, while complementary calculations were performed for the others as described in Online Appendix Table 1. For 9 studies, seroprevalence estimates were corrected for test performance using the Gladen–Rogan formula (Online Appendix Table 4). Median IFR in all elderly for all 11 high-income countries was 4.5% (range 2.5–16.7%). Of the 13 included estimates, the IFR in community-dwelling elderly was > 30% lower than the IFR among all elderly in 10/13 and > 50% lower in 1/13.

With 5% relative monthly seroreversion, median IFR in community-dwelling elderly in 11 high-income countries was 2.2% (4.0% in all elderly—see details on seroreversion analyses in Online Appendix Table 5). Online Appendix Table 6 shows calculations with later cut-off for counting deaths.

The median IFR was 2.7% upon excluding 3 studies where the selected time point with highest seroprevalence was not the latest available (seroprevalence had declined in the latest timepoint) [56–58]. The median IFR was 2.8% in 15 countries when applying the non-peer-reviewed pre-specified original eligibility criteria (including convenience samples, including 2 middle-income countries, and correcting for unmeasured antibodies).

### IFR in the elderly and proportion > 85 years

**Fig. 2:**
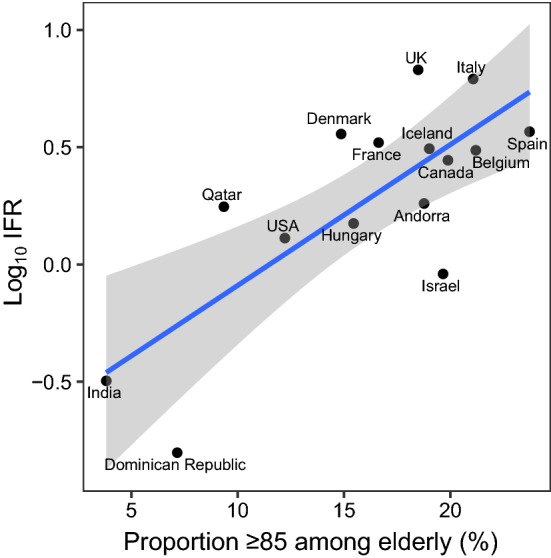
Infection fatality rate in community-dwelling elderly, corrected for unmeasured antibody types, plotted against the proportion of people ≥ 85 years old among the elderly. Log_10_ IFR: logarithm (with base 10) of the infection fatality rate. The “elderly” group is defined by the primary cutoff for each location. E.g. for the USA 2% of the population is ≥ 85, 16.5% of the population is ≥ 65, and the proportion is 2/16.5. Imputation done for Tamil Nadu, India, with country-level proportion of persons ≥ 85 years old among elderly

IFR tended to increase with larger proportions of people ≥ 85 years old (Fig. 2). A regression of logIFR against the proportion of people ≥ 85 years old had a slope of 0.03 (*p* = 0.17), and suggested an IFR in community-dwelling elderly of 1.21%, 1.79%, and 3.89% when the proportion of people > 85 in the elderly group was 5%, 10%, and 20%, respectively. Using the set of studies in the pre-specified original eligibility criteria, the slope was 0.06 (*p* = 0.001), with IFR 0.41%, 0.81%, and 3.25% for the respective proportions.

## Discussion

The IFR of COVID-19 in elderly was found to vary widely at locations where seroprevalence studies have enrolled many elderly individuals. IFR in community-dwelling elderly was consistently lower than in elderly overall. In countries where nursing homes are widely used, the difference was very substantial.

Early estimates of case fatality rate (CFR, ratio of deaths divided by *documented* infections) [59–61] were very high. However, infected individuals far exceeded documented cases [62]. IFR is much lower than CFR. Our work represents the only effort to-date to synthesize data on age-stratified IFR estimates using a detailed prespecified and registered protocol, with justifications whenever further decisions had to be made. See Online Appendix Text 1 for comparison of our IFR estimates against previous relevant work [4, 5, 63, 64]. Our estimates, when inferred for the USA, are distinctly lower than the current Centers for Disease Control and Prevention best planning scenario [63]. IFR estimates in the elderly are still extremely higher than IFR in younger age groups (Online Appendix Text 1).

Substantial true heterogeneity is expected since IFR is situation- and population-dependent. Both age distribution and other characteristics of people within the elderly stratum vary between different countries. Moreover, criteria for coding COVID-19 deaths may have varied across countries. Under- and over-counting of COVID-19 deaths probably occurred even in countries with advanced health systems.

Observed differences in IFR between community-dwelling elderly and elderly overall are consistent with previous findings that beyond age, comorbid conditions and frailty drive COVID-19 mortality [10]. People living in institutions account for many COVID-19 deaths [65], thus a location’s overall IFR is largely dependent on how these facilities were afflicted [5]. Spread in nursing homes was disproportionately high in the first wave [8]. IFR in residents can be much higher than the IFR in community-dwelling elderly. Seroprevalence studies of long-term care populations in Spain (Madrid), northern Italy, UK and Brazil in early phases of the epidemic found prevalence of 55%, 41%, 33%, and 11.5%, respectively [66–70], i.e. several fold higher than prevalence in the general community populations in these locations. Under-estimation of infections due to seroreversion may also be prominent in such studies [60]. Large diversity in seroprevalence can exist between facilities, e.g. in Brazil [69] the seroprevalence was 100% and 76%, respectively in 2 nursing homes that had outbreaks and 0% or close to 0% in another 13. The IFR in that study was 25%, a reasonable estimate for nursing homes with rather frail populations, as corroborated also by other investigators [11]. IFR may be much lower in facilities where residents in overall good health plan to spend many years of their life; but extremely high in palliative care facilities. Furthermore, our estimates of IFR among community-dwelling elderly are probably inflated, because in some countries not all long-term care facilities were included in the count of long-term care deaths.

Nursing home deaths decreased markedly over time [65] in most high-income countries. This change may have decreased IFR markedly after the first wave. Improved treatments and less use of harmful treatments may also have decreased IFR substantially in late 2020 and 2021 [71, 72]. Other investigators estimated in 2020 a global IFR of 0.11% [73]. Vaccines that are more effective in protecting against death rather than infection are also expected to have decreased IFR in 2021. New variants in 2021 may be associated with further lower IFR. E.g., in countries with extensive testing such as the UK, when the delta variant spread widely even CFR remained ~ 0.3% [74]. Preliminary data on the omicron variant in late 2021 suggest even lower severity [75].

Our analysis has several limitations. First, seroprevalence estimates among elderly reported by the included studies could over- or underestimate the proportion infected. We explored adjusted estimates accounting for 1–10% relative seroreversion per month; however, higher seroreversion is likely [21, 22, 32]. Higher seroreversion will affect more prominently studies carried out later in the pandemic. Also, the current estimates do not fully account for the unknown share of people who may have tackled the infection without generating detectable serum/plasma antibodies (e.g., by mucosal, innate, or cellular [T cell] immune mechanisms) [76–80]. Sensitivity estimates for antibody assays typically use positive controls from symptomatic individuals with clinically manifest infection; sensitivity may be lower for asymptomatic infections. All seroprevalence studies may have substantial residual biases despite whatever adjustments [6]. Even well-designed general population studies may specifically fail to reach and recruit highly vulnerable populations, e.g. disadvantaged groups, immigrants, homeless, and other people at high exposure risks and poor health. For studies carried out in the US, we prespecified an eligibility criterion to adjust for race/ethnicity, which we believe acts to mitigate related biases. COVID-19-related racial/ethnic differences were expected to be stronger in the USA than in other high-income countries, and racial/ethnic diversity is far more prominent in the USA (e.g. 57.8% of the population identify as non-Hispanic white, while the proportion is over 90% in Denmark and 85% in France).

Second, numbers of deaths may be biased for various reasons [3] leading to potential under- or over-counting. Underreporting of deaths is debated specifically for India (e.g., [81]). Online Appendix Text 2 discusses issues related to India and other non-high income countries and explains why simple excess death estimates should not be used to calculate IFR [82].

Sensitivity analysis that extended with 1 week the cutoff for counting deaths showed a negligible change in estimated median IFR. Most studies included in our analysis had been performed during periods at or after the end of the first wave. Some studies performed sampling for several months, which introduces further uncertainty. However, typically the sampling covered periods with few fatalities.

Third, bias exists in seroprevalence studies, mortality statistics, and even population statistics. However, assessments of risk of bias are far from straightforward, as illustrated by discrepant assessments of these seroprevalence studies by other teams [6]. Moreover, estimates cited here have uncertainty going beyond the presented 95% CIs. Others, e.g., Campbell and Gustafson [83], have proposed models that incorporate additional uncertainty.

Fourth, even among high-income countries, our set of eligible surveys tends to include mostly data from countries with higher death rates, thus possibly also higher IFR. More prominently, our analysis includes limited data from Asia and no data from Africa. Consideration of age strata diminishes this representativeness bias, but cannot eliminate it. E.g., most countries not represented may have a shift towards lower ages within the elderly stratum. This translates to lower IFR. Moreover, high-income countries analyzed here have population prevalence of obesity 1.5–3-fold higher than the global prevalence (13%); other major risk factors for poor COVID-19 outcome (smoking, diabetes, cardiovascular disease, immunosuppression) [9] are also far more common in the high-income countries included in our analysis than the global average. Global IFR may thus be substantially lower in both the elderly and the lower age strata than estimates presented herein.

Fifth, many complementary pieces of information were needed beyond the systematic search for seroprevalence studies. Some decisions made in this process, e.g., the eligibility criterion of having many elderly participants, could be described as arbitrary. This rule was introduced for feasibility and validity, since small samples are largely uninformative (given the huge uncertainty). The rule tends to prefer studies in populations with old age pyramids and many frail individuals who survive into late age with many comorbidities.

This overview synthesis finds a consistently lower IFR of COVID-19 in community-dwelling elderly than in elderly overall, a difference which is substantial in countries with many long-term care facilities. The estimates presented here may help inform public health policy decisions.

## Supplementary Information

Below is the link to the electronic supplementary material.

Supplementary file1 (DOCX 418 KB)

## Data Availability

The protocol, data, and code used for this analysis will be made available at the Open Science Framework upon publication: https://osf.io/47cgb.

## References

[1] Ioannidis JPA, Axfors C, Contopoulos-Ioannidis DG (2020). Population-level COVID-19 mortality risk for non-elderly individuals overall and for non-elderly individuals without underlying diseases in pandemic epicenters. Environ Res.

[2] Comas-Herrera A, Zalakaín J, Lemmon E, Henderson D, Litwin C, Hsu A, et al. Mortality associated with COVID-19 in care homes: international evidence. Article in LTCcovid.org, International Long-Term Care Policy Network, CPEC-LSE. 14 October2020.

[3] Ioannidis JPA (2021). Infection fatality rate of COVID-19 inferred from seroprevalence data. Bull World Health Organ.

[4] Levin AT, Hanage WP, Owusu-Boaitey N, Cochran KB, Walsh SP, Meyerowitz-Katz G (2020). Assessing the age specificity of infection fatality rates for COVID-19: systematic review, meta-analysis, and public policy implications. Eur J Epidemiol.

[5] O’Driscoll M, Dos Santos GR, Wang L, Cummings DAT, Azman AS, Paireau J, et al. Age-specific mortality and immunity patterns of SARS-CoV-2. Nature. 2021;590:140–5. 10.1038/s41586-020-2918-0 33137809

[6] Ioannidis JPA (2021). Reconciling estimates of global spread and infection fatality rates of COVID-19: an overview of systematic evaluations. Eur J Clin Investig.

[7] Arora RK, Joseph A, Van Wyk J, Rocco S, Atmaja A, May E (2020). SeroTracker: a global SARS-CoV-2 seroprevalence dashboard. Lancet Infect Dis.

[8] Ioannidis JPA (2021). Precision shielding for COVID-19: metrics of assessment and feasibility of deployment. BMJ Glob Health.

[9] Williamson EJ, Walker AJ, Bhaskaran K, Bacon S, Bates C, Morton CE (2020). Factors associated with COVID-19-related death using OpenSAFELY. Nature.

[10] Mak JKL, Kuja-Halkola R, Wang Y, Hägg S, Jylhävä J (2021). Frailty and comorbidity in predicting community COVID-19 mortality in the U.K. Biobank: the effect of sampling. J Am Geriatr Soc.

[11] Arons MM, Hatfield KM, Reddy SC, Kimball A, James A, Jacobs JR (2020). Presymptomatic SARS-CoV-2 infections and transmission in a skilled nursing facility. N Engl J Med.

[12] Franceschi VB, Santos AS, Glaeser AB, Paiz JC, Caldana GD, Machado Lessa CL (2020). Population-based prevalence surveys during the Covid-19 pandemic: a systematic review. Rev Med Virol.

[13] Chen X, Chen Z, Azman AS, Deng X, Sun R, Zhao Z (2021). Serological evidence of human infection with SARS-CoV-2: a systematic review and meta-analysis. Lancet Glob Health.

[14] Ioannidis JPA. Precision shielding for COVID-19: metrics of assessment and feasibility of deployment. BMJ Glob Health. 2021;6(1):e004614. 10.1136/bmjgh-2020-004614. PMID: 33514595; PMCID: PMC7849322. 10.1136/bmjgh-2020-004614 PMC7849322 33514595

[15] Patel EU, Bloch EM, Grabowski MK, Goel R, Lokhandwala PM, Brunker PAR (2019). Sociodemographic and behavioral characteristics associated with blood donation in the United States: a population-based study. Transfusion.

[16] Sohn H (2017). Racial and ethnic disparities in health insurance coverage: dynamics of gaining and losing coverage over the life-course. Popul Res Policy Rev.

[17] Qeadan F, VanSant-Webb E, Tingey B, Rogers TN, Brooks E, Mensah NA (2021). Racial disparities in COVID-19 outcomes exist despite comparable Elixhauser comorbidity indices between Blacks, Hispanics, Native Americans, and Whites. Sci Rep.

[18] Ahmad F, Cisewski JA, Miniño A, Anderson RN (2021). Provisional mortality data—United States, 2020. MMWR Morb Mortal Wkly Rep.

[19] Xu JJ, Chen JT, Belin TR, Brookmeyer RS, Suchard MA, Ramirez CM (2021). Racial and ethnic disparities in years of potential life lost attributable to COVID-19 in the United States: an analysis of 45 states and the district of Columbia. Int J Environ Res Public Health.

[20] Munn Z, Moola S, Lisy K, Riitano D, Tufanaru C (2015). Methodological guidance for systematic reviews of observational epidemiological studies reporting prevalence and cumulative incidence data. Int J Evid Based Healthc.

[21] Shioda K, Lau MSY, Kraay ANM, Nelson KN, Siegler AJ, Sullivan PS, et al. Estimating the Cumulative Incidence of SARS-CoV-2 infection and the infection fatality ratio in light of waning antibodies. Epidemiology. 2021;32(4):518–24. 10.1097/EDE.0000000000001361. 10.1097/EDE.0000000000001361 PMC8162228 33935138

[22] The COVID-19 Community Research Partnership Study Group. Duration of SARS-CoV-2 sero-positivity in a large longitudinal sero-surveillance cohort: the COVID-19 Community Research Partnership. BMC Infect Dis. 2021;21:889. 10.1186/s12879-021-06517-6. 10.1186/s12879-021-06517-6 PMC8404407 34461847

[23] Post N, Eddy D, Huntley C, van Schalkwyk MCI, Shrotri M, Leeman D (2020). Antibody response to SARS-CoV-2 infection in humans: a systematic review. PLoS ONE.

[24] Faes C, Abrams S, Van Beckhoven D, Meyfroidt G, Vlieghe E, Hens N (2020). Time between symptom onset, hospitalisation and recovery or death: statistical analysis of Belgian COVID-19 patients. Int J Environ Res Public Health.

[25] Comas-Herrera A, Zalakaín J, Litwin C, Hsu A, Lemmon E, Henderson D, et al. Mortality associated with COVID-19 in care homes: early international evidence. Article in LTCcovid.org, International Long-Term Care Policy Network, CPEC-LSE, 26 June2020

[26] Office for National Statistics. Deaths involving COVID-19 in the care sector, England and Wales. Office for National Statistics; 2020. https://www.ons.gov.uk/peoplepopulationandcommunity/birthsdeathsandmarriages/deaths/datasets/deathsinvolvingcovid19inthecaresectorenglandandwales. Updated 3 July 2020.

[27] Rogan WJ, Gladen B (1978). Estimating prevalence from the results of a screening test. Am J Epidemiol.

[28] Garcia-Basteiro AL, Moncunill G, Tortajada M, Vidal M, Guinovart C, Jiménez A (2020). Seroprevalence of antibodies against SARS-CoV-2 among health care workers in a large Spanish reference hospital. Nat Commun.

[29] R Core Team. R: A language and environment for statistical computing. Foundation for Statistical Computing, Vienna, Austria; 2020. https://www.R-project.org/.

[30] Ioannidis JP, Patsopoulos NA, Rothstein HR (2008). Reasons or excuses for avoiding meta-analysis in forest plots. BMJ.

[31] Deeks J, Higgins J, Altman D. Analysing data and undertaking meta-analyses. Section 10.10 Heterogeneity. Cochrane handbook for systematic reviews of interventions version 62 (updated February 2021). Cochrane; 2021. www.training.cochrane.org/handbook.

[32] Pérez-Olmeda M, Saugar JM, Fernández-García A, Pérez-Gómez B, Pollán M, Avellón A (2021). Evolution of antibodies against SARS-CoV-2 over seven months: experience of the nationwide seroprevalence ENE-COVID Study in Spain. medRxiv.

[33] Royo-Cebrecos C, Vilanova D, López J, Arroyo V, Pons M, Francisco G (2021). Mass SARS-CoV-2 serological screening, a population-based study in the principality of Andorra. Lancet Reg Health Eur.

[34] Public Health Ontario. COVID-19 seroprevalence in Ontario: March 27, 2020 to June 30, 2020. https://www.publichealthontario.ca/. 2020.

[35] Espenhain L, Tribler S, Sværke Jørgensen C, Holm Hansen C, Wolff Sönksen U, Ethelberg S (2021). Prevalence of SARS-CoV-2 antibodies in Denmark: nationwide, population-based seroepidemiological study. Eur J Epidemiol.

[36] Paulino-Ramirez R, Báez AA, Degaudenzi AV, Tapia L. Seroprevalence of specific antibodies against SARS-CoV-2 from hotspot communities in the dominican republic. Amer J Tropical Med Hygiene 2020;103(6):2343–46. 10.4269/ajtmh.20-0907. 10.4269/ajtmh.20-0907 PMC7695105 33094710

[37] Warszawski J, Meyer L, Franck J-E, Rahib D, Lydié N, Gosselin A, et al. Trends in social exposure to SARS-Cov-2 in France. Evidence from the national socio-epidemiological cohort – EPICOV. medRxiv. 2021;2021.10.25.21265456. 10.1371/journal.pone.0267725 PMC9132278 35613100

[38] Carrat F, de Lamballerie X, Rahib D, Blanché H, Lapidus N, Artaud F, Kab S, Renuy A, Szabo de Edelenyi F, Meyer L, Lydié N, Charles MA, Ancel PY, Jusot F, Rouquette A, Priet S, Saba PM, Fourié VT, Lusivika-Nzinga C, Nicol J, Legot S, Druesne-Pecollo N, Esseddik Y, Lai C, Gagliolo JM, Deleuze JF, Bajos N, Severi G, Touvier M, Zins M. Antibody status and cumulative incidence of SARS-CoV-2 infection among adults in three regions of France following the first lockdown and associated risk factors: a multicohort study. Int J Epidemiol 2021;50(5):1458–72. 10.1093/ije/dyab110. 10.1093/ije/dyab110 PMC8344948 34293141

[39] Merkely B, Szabó AJ, Kosztin A, Berényi E, Sebestyén A, Lengyel C (2020). Novel coronavirus epidemic in the Hungarian population, a cross-sectional nationwide survey to support the exit policy in Hungary. GeroScience.

[40] Gudbjartsson DF, Norddahl GL, Melsted P, Gunnarsdottir K, Holm H, Eythorsson E (2020). Humoral immune response to SARS-CoV-2 in Iceland. N Engl J Med.

[41] Murhekar MV, Bhatnagar T, Selvaraju S, Rade K, Saravanakumar V, Thangaraj JW, Kumar MS, Shah N, Sabarinathan R, Turuk A, Anand PK, Asthana S, Balachandar R, Bangar SD, Bansal AK, Bhat J, Chakraborty D, Rangaraju C, Chopra V, Das D, Deb AK, Devi KR, Dwivedi GR, Salim Khan SM, Haq I, Kumar MS, Laxmaiah A, Mahapatra MA, Mitra A, Nirmala AR, Pagdhune A, Qurieshi MA, Ramarao T, Sahay S, Sharma YK, Shrinivasa MB, Shukla VK, Singh PK, Viramgami A, Wilson VC, Yadav R, Girish Kumar CP, Luke HE, Ranganathan UD, Babu S, Sekar K, Yadav PD, Sapkal GN, Das A, Das P, Dutta S, Hemalatha RK, Kumar A, Narain K, Narasimhaiah S, Panda S, Pati S, Patil S, Sarkar K, Singh S, Kant R, Tripathy S, Toteja GS, Babu GR, Kant S, Muliyil JP, Pandey RM, Sarkar S, Singh SK, Zodpey S, Gangakhedkar RR, Reddy DCS, Bhargava B. Prevalence of SARS-CoV-2 infection in India: findings from the national serosurvey May-June 2020. Indian J Med Res 152(1):2020;48. 10.4103/ijmr.IJMR_3290_20. 10.4103/ijmr.IJMR_3290_20 PMC7853249 32952144

[42] Malani A, Ramachandran S, Tandel V, Parasa R, Sudharshini S, Prakash V, et al. SARS-CoV-2 seroprevalence in Tamil Nadu in October-November 2020. medRxiv. 2021;21250949.

[43] Reicher S, Ratzon R, Ben-Sahar S, Hermoni-Alon S, Mossinson D, Shenhar Y (2021). Nationwide seroprevalence of antibodies against SARS-CoV-2 in Israel. Eur J Epidemiol.

[44] Istat - Istituto Nazionale di Statistica, Ministero della Salute. PRIMI RISULTATI DELL’INDAGINE DI SIEROPREVALENZA SUL SARS-CoV-2. 2020. https://www.istatit/it/files/2020/08/ReportPrimiRisultatiIndagineSieropdf.

[45] Šmigelskas K, Petrikonis K, Kasiulevičius V, Kalėdienė R, Jakaitienė A, Kaselienė S, et al. SARS-CoV-2 Seroprevalence in Lithuania: Results of National Population Survey. Acta medica Lituanica. 2021;28(1).Vos ERA, van Boven 10.15388/Amed.2020.28.1.2 PMC8311832 34393628

[46] Vos ERA, van Boven M, den Hartog G, Backer JA, Klinkenberg D, van Hagen CCE (2021). Associations between measures of social distancing and SARS-CoV-2 seropositivity: a nationwide population-based study in the Netherlands. medRxiv.

[47] Ministerio de Sanidad, III IdSC. ESTUDIO ENE-COVID: CUARTA RONDA. ESTUDIO NACIONAL DE SERO-EPIDEMIOLOGÍA DE LA INFECCIÓN POR SARS-COV-2 EN ESPAÑA. 15 DE DICIEMBRE DE 2020. 2021. https://www.mscbsgobes/gabinetePrensa/notaPrensa/pdf/1512151220163348113pdf; https://www.portalcneisciiies/enecovid19/informes/informe_cuarta_rondapdf.

[48] Ward H, Cooke GS, Atchison C, Whitaker M, Elliott J, Moshe M (2021). Prevalence of antibody positivity to SARS-CoV-2 following the first peak of infection in England: serial cross-sectional studies of 365,000 adults. Lancet Reg Health Eur.

[49] Kalish H, Klumpp-Thomas C, Hunsberger S, Baus HA, Fay MP, Siripong N (2021). Undiagnosed SARS-CoV-2 seropositivity during the first 6 months of the COVID-19 pandemic in the United States. Sci Transl Med.

[50] Herzog S, De Bie J, Abrams S, Wouters I, Ekinci E, Patteet L (2021). Seroprevalence of IgG antibodies against SARS coronavirus 2 in Belgium—a serial prospective cross-sectional nationwide study of residual samples (March–October 2020). medRxiv.

[51] Saeed S, Drews SJ, Pambrun C, Yi QL, Osmond L, O'Brien SF (2021). SARS-CoV-2 seroprevalence among blood donors after the first COVID-19 wave in Canada. Transfusion.

[52] Charlton CL, Nguyen LT, Bailey A, Fenton J, Plitt SS, Marohn C (2021). Pre-vaccine positivity of SARS-CoV-2 antibodies in Alberta, Canada during the first two waves of the COVID-19 pandemic. Microbiol Spectrum.

[53] Pedersen OB, Nissen J, Dinh KM, Schwinn M, Kaspersen KA, Boldsen JK, et al. Severe acute respiratory syndrome coronavirus 2 (SARS-CoV-2) infection fatality rate among elderly danes: a cross-sectional study on retired blood donors. Clin Infect Dis. 2021;73:e2962–9. 10.1093/cid/ciaa1627 PMC7665387 33103182

[54] Abu-Raddad LJ, Chemaitelly H, Ayoub HH, Al Kanaani Z, Al Khal A, Al Kuwari E (2021). Characterizing the qatar advanced-phase SARS-CoV-2 epidemic. Sci Rep.

[55] UK Biobank. UK Biobank SARS-CoV-2 serology study. 16th September 2020. https://www.ukbiobank.ac.uk/.

[56] Public Health England, Joint Biosecurity Centre, NHS Test and Trace. Weekly Coronavirus Disease 2019 (COVID-19) Surveillance Report. Summary of COVID-19 surveillance systems. Year: 2020. Week: 32. 2020. https://assets.publishing.service.gov.uk/.

[57] Hughes EC, Amat JAR, Haney J, Parr YA, Logan N, Palmateer N (2021). Severe acute respiratory syndrome coronavirus 2 serosurveillance in a patient population reveals differences in virus exposure and antibody-mediated immunity according to host demography and healthcare setting. J Infect Dis.

[58] Anand S, Montez-Rath M, Han J, Bozeman J, Kerschmann R, Beyer P (2020). Prevalence of SARS-CoV-2 antibodies in a large nationwide sample of patients on dialysis in the USA: a cross-sectional study. Lancet.

[59] The Novel Coronavirus Pneumonia Emergency Response Epidemiology Team (2020). Vital surveillances: the epidemiological characteristics of an outbreak of 2019 novel coronavirus diseases (COVID-19)—China, 2020. China CDC Wkly.

[60] Onder G, Rezza G, Brusaferro S (2020). Case-Fatality rate and characteristics of patients dying in relation to COVID-19 in Italy. JAMA.

[61] Thompson C, Baumgartner J, Pichardo C (2020). COVID-19 outbreak—New York City, February 29-June 1, 2020. MMWR Morb Mortal Wkly Rep.

[62] Bendavid E, Mulaney B, Sood N, Shah S, Bromley-Dulfano R, Lai C (2021). COVID-19 antibody seroprevalence in Santa Clara County, California. Int J Epidemiol.

[63] United States Centers for Disease Control and Prevention. COVID-19 pandemic planning scenarios 2021 [updated March 19, 2021. Available from: https://www.cdc.gov/coronavirus/2019-ncov/hcp/planning-scenarios.html.

[64] Brazeau N, Verity R, Jenks S (2020). COVID-19 infection fatality ratio: estimates from seroprevalence.

[65] Ioannidis JPA, Axfors C, Contopoulos-Ioannidis DG (2021). Second versus first wave of COVID-19 deaths: shifts in age distribution and in nursing home fatalities. Environ Res.

[66] Candel FJ, Barreiro P, San Román J, Del Mar Carretero M, Sanz JC, Pérez-Abeledo M (2021). The demography and characteristics of SARS-CoV-2 seropositive residents and staff of nursing homes for older adults in the community of Madrid: the SeroSOS study. Age Ageing.

[67] Vena A, Berruti M, Adessi A, Blumetti P, Brignole M, Colognato R (2020). Prevalence of antibodies to SARS-CoV-2 in Italian adults and associated risk factors. J Clin Med.

[68] Krutikov M, Palmer T, Tut G, Fuller C, Shrotri M, Williams H (2021). Incidence of SARS-CoV-2 infection according to baseline antibody status in staff and residents of 100 long-term care facilities (VIVALDI): a prospective cohort study. Lancet Healthy longev.

[69] Barros ENC, Valle APD, Braga PE, Viscondi JYK, Fonseca A, Vanni T (2021). COVID-19 in long-term care facilities in Brazil: serological survey in a post-outbreak setting. Rev Inst Med Trop Sao Paulo.

[70] Krutikov M, Palmer T, Tut G, Fuller C, Azmi B, Giddings R, et al. Prevalence and duration of detectable SARS-CoV-2 nucleocapsid antibodies in staff and residents of long-term care facilities over the first year of the pandemic (VIVALDI study): prospective cohort study in England. The Lancet Healthy Longevity. 2022;3(1):e13. 10.1016/S2666-7568(21)00282-8 PMC8676418 34935001

[71] The RECOVERY Collaborative Group (2020). Dexamethasone in hospitalized patients with Covid-19. New Engl J Med.

[72] Axfors C, Schmitt AM, Janiaud P, van’t Hooft J, Abd-Elsalam S, Abdo EF, et al. Mortality outcomes with hydroxychloroquine and chloroquine in COVID-19 from an international collaborative meta-analysis of randomized trials. Nat Commun. 2021;12(1):2349. 10.1038/s41467-021-22446-z. 10.1038/s41467-021-22446-z PMC8050319 33859192

[73] Ayoub HH, Mumtaz GR, Seedat S, Makhoul M, Chemaitelly H, Abu-Raddad LJ (2021). Estimates of global SARS-CoV-2 infection exposure, infection morbidity, and infection mortality rates in 2020. Glob Epidemiol.

[74] Public Health England COVID-19 Epidemiology Cell. COVID-19 confirmed deaths in England (to 31 August 2021): report. Updated 12 October 2021. https://www.gov.uk/government/publications/covid-19-reported-sars-cov-2-deaths-in-england/covid-19-confirmed-deaths-in-england-to-31-august-2021-report#case-fatality-risk. 2021.

[75] Imperial College COVID-19 response Team. Ferguson N, Ghni A, Hinsley W, Volz E. Report 50: Hospitalization risk for Omicron cases in England. 22 December 2021. 10.25561/930352021.

[76] Kikkert M (2020). Innate Immune Evasion by Human Respiratory RNA Viruses. J Innate Immun.

[77] Krammer F (2019). The human antibody response to influenza A virus infection and vaccination. Nat Rev Immunol.

[78] Cervia C, Nilsson J, Zurbuchen Y, Valaperti A, Schreiner J, Wolfensberger A (2021). Systemic and mucosal antibody responses specific to SARS-CoV-2 during mild versus severe COVID-19. J Allergy Clin Immunol.

[79] Gallais F, Velay A, Nazon C, Wendling M-J, Partisani M, Sibilia J (2021). Intrafamilial exposure to SARS-CoV-2 associated with cellular immune response without seroconversion, France. Emerg Infect Dis.

[80] Sekine T, Perez-Potti A, Rivera-Ballesteros O, Strålin K, Gorin JB, Olsson A (2020). Robust T cell immunity in convalescent individuals with asymptomatic or mild COVID-19. Cell.

[81] Deshmukh Y, Suraweera W, Tumbe C, Bhowmick A, Sharma S, Novosad P (2021). Excess mortality in India from June 2020 to June 2021 during the COVID pandemic: death registration, health facility deaths, and survey data. medRxiv.

[82] Laxminarayan R, Chandra Mohan B, Vinay TG, Arjun Kumar KV, Wahl B, Lewnard JA (2021). SARS-CoV-2 infection and mortality during the first epidemic wave in Madurai, south India: a prospective, active surveillance study. Lancet Infect Dis.

[83] Campbell H, Gustafson P. Inferring the COVID-19 infection fatality rate in the community-dwelling population: a simple Bayesian evidence synthesis of seroprevalence study data and imprecise mortality data. Epidemiol Infect. 2021;149:E243. 10.1017/S0950268821002405

