# Supplementary material for: Infection fatality rate of COVID-19 in community-dwelling elderly populations
Source: Eur J Epidemiol. 2022 Mar 20;37(3):235–49. doi: 10.1007/s10654-022-00853-w (PMC8934243; doi:10.1007/s10654-022-00853-w)
Supplement: Supplementary file 1 — Supplementary file1 (DOCX 418 KB) [file 10654_2022_853_MOESM1_ESM.docx]

## **APPENDIX**

**Infection fatality rate of COVID-19 in community-dwelling elderly populations**

Cathrine Axfors^1^, John P A Ioannidis^1,2^

^1^Meta-Research Innovation Center at Stanford (METRICS), Stanford University, Stanford, California, USA

^2^Departments of Medicine, of Epidemiology and Population Health, of Biomedical Data Science, and of Statistics, Stanford University, Stanford, California, USA

Correspondence to: John P A Ioannidis, Stanford Prevention Research Center, Medical School Office Building, Room X306, 1265 Welch Road, Stanford CA 94305, USA

Tel: 650-7045584; E-mail: [jioannid@stanford.edu](mailto:jioannid@stanford.edu)

1. **Appendix Figure 1.** Flowchart of the search and selection process.
2. **Appendix Table 1**. Amendments to protocol
3. **Appendix Table 2**. Sources for COVID-19 mortality statistics and population statistics.
4. **Appendix Table 3a.** Reports not included, main analysis (complementary search for explicitly national-level general population studies without high risk of bias and with at least 500 participants aged ≥70 years).
5. **Appendix Table 3b.** Reports not included, sensitivity analysis (original search for studies that sampled or potentially sampled ≥1000 participants aged ≥70 years).
6. **Appendix Table 4.** Seroprevalence estimates corrected for test performance using the Gladen-Rogan formula.
7. **Appendix Table 5.** Uncorrected and corrected (for unmeasured antibody types or for seroreversion) infection fatality rate in community-dwelling elderly
8. **Appendix Table 6**. Sensitivity analysis with a later cutoff for cumulative COVID-19 mortality (study midpoint plus two weeks instead of plus one week).
9. **Appendix Text 1.** Comparison against other studies estimating IFR.
10. **Appendix Text 2.** India and non-high-income countries and excess deaths.

**Appendix Figure 1.** Flowchart of the search and selection process.


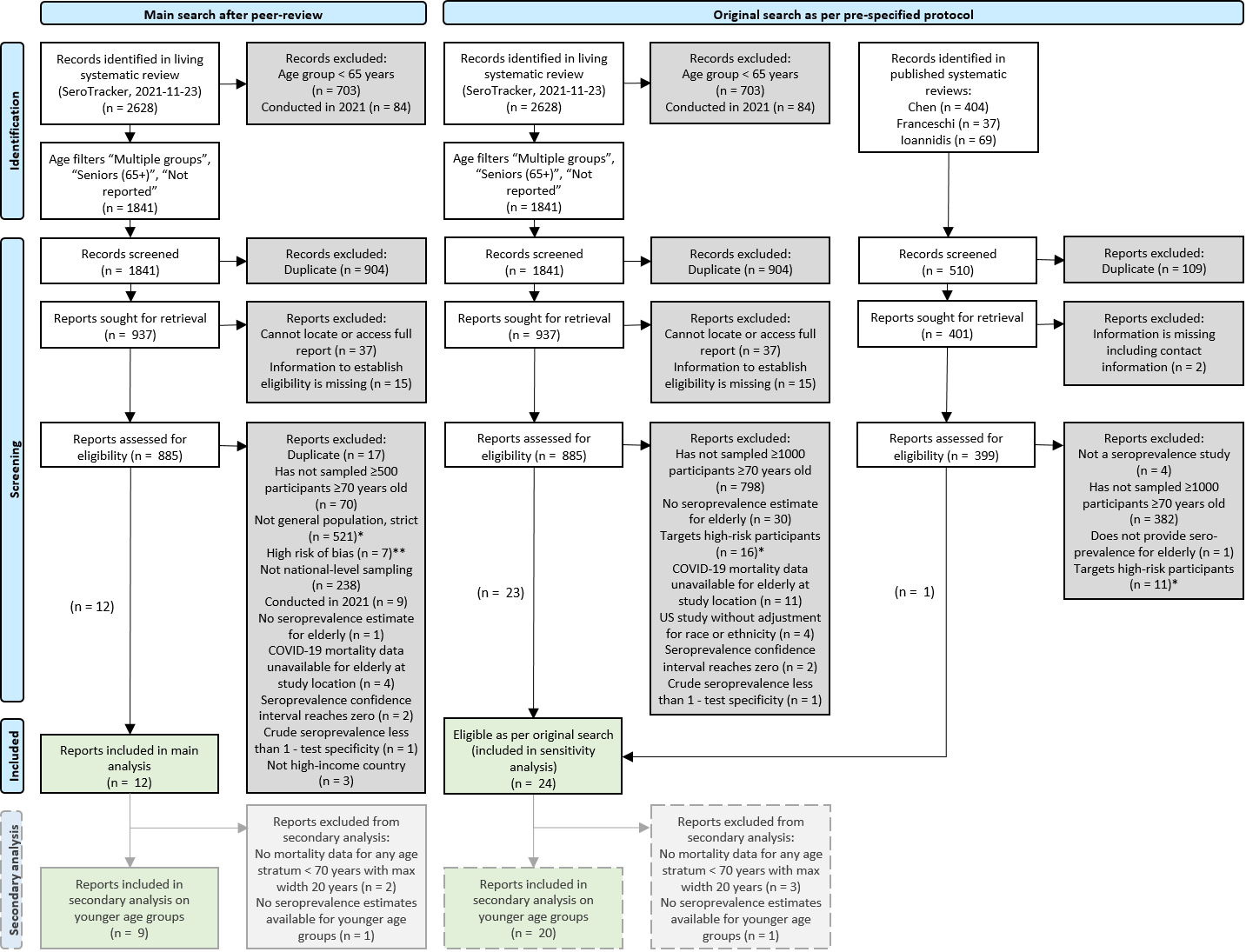


Systematic reviews used as sources: SeroTracker (7); Chen et al (12), Franceschi et al (11), Ioannidis (13).

SeroTracker is a living systematic review with daily screening of published articles (MEDLINE, Embase, Web of Science, Cochrane), preprints (medRxiv, bioRxiv), government reports, and news articles. Data on identified seroprevalence studies are available for downloading at www.serotracker.com including study characteristics such as sampled age groups.

*High-risk participants (original search, now sensitivity analysis): healthcare workers, essential workers, contacts to COVID-19 patients, or residual patient samples where COVID-19-related visits were not excluded. A stricter criterion was used for general population samples in the main analysis, based on SeroTracker categories of “Household and community samples” and “Multiple general populations”. ** Risk of bias according to assessments by the SeroTracker team.

In the final sample (17 reports; 14 reports in secondary analysis), one study presented data on two eligible surveys.

**Appendix Table 1**. Amendments to protocol.^*^

| **Manuscript section** | **Amendment** | **Rationale** |
| --- | --- | --- |
| Eligibility criteria | In the original protocol, we aimed to include studies on SARS-CoV-2 seroprevalence that had sampled at least 1000 participants aged ≥70 years in the location and/or setting of interest, provided an estimate of seroprevalence for elderly people, explicitly aimed to generate samples reflecting the general population, and were conducted at a location for which there is official data available on the proportion of cumulative COVID-19 deaths among elderly (with a cutoff placed between 60-70 years; e.g., eligible cutoffs were ≥70, ≥65, or ≥60, but not ≥75 or ≥55).  Following comments from peer-reviewers, we also included national-level general population studies (based on the SeroTracker categories of “Household and community samples” and “Multiple general populations”) without high risk of bias (reported by the SeroTracker team using the Joanna Briggs Institute Critical Appraisal Tool for Prevalence Studies) and with at least 500 participants aged ≥70 years; we excluded samples of patient cohorts, insurance applicants, blood donors, and workers (that were intended for inclusion in the original protocol); and we excluded studies from middle-income countries.  A sensitivity analysis was added that considers the original inclusion criteria. | Following comments arising during peer review |
| Eligibility criteria | Added eligibility criterion: excluding seroprevalence studies that sampled participants after Dec 31, 2020. | IFR may be substantially affected (mostly shifting downwards) by vaccination (especially with prioritization among the most aged and frail individuals), better treatment, and new variants emerging and becoming dominant in 2021. We located only one study with sampling in 2021 (Office of National Statistics, United Kingdom, February 2021). |
| Eligibility criteria | Added eligibility criterion: excluding seroprevalence studies where crude seroprevalence is less than 1- test specificity and/or the 95% confidence interval of the seroprevalence goes to 0%. | Following comments arising during peer review, since the seroprevalence estimate would be extremely uncertain. |
| Eligibility criteria | Added clarification: For studies with several sampled subregions of a country we applied the criterion of ≥1000 sampled individuals ≥70 years for each subregion, unless (1) the sampling locations were dispersed across the country so as to form a reasonable representation of the entire country, in which case the country was considered the location; or (2) an aggregate seroprevalence estimate was available *and* age-stratified COVID-19 mortality statistics and population statistics were available for each subregion, in which case the regions combined were considered the location. | Clarification was necessary for this eligibility criterion in the case of studies with several sampled subregions. As far as possible, we strived to combine subregional sampling with the respective COVID-19 mortality and population statistics. |
| Data extraction | Added clarification: We extracted the number of deaths for the primary date from official reports or Worldometer (or, if the location was not present on Worldometer, Wikipedia), whichever presented the higher number. For official reports, we preferred the latest updated dataset of deaths by date of occurrence (not date of reporting) if available, otherwise situational reports. | Minor discrepancies were observed between sources, possibly attributable to retrospectively reported deaths, why the higher number was chosen.  Latest available datasets were preferred to situational reports because of the addition of retrospectively reported deaths. |
| Data extraction | If the number of deaths occurring in nursing homes (i.e., excluding deaths occurring in hospitals) was the only available estimate, we calculated the total number of deaths in nursing home residents with a correction (by multiplying with the median of available ratios of deaths in nursing homes to deaths of nursing home residents in the International Long Term Care Policy Network report of October 14 of countries in the same continent). | In order to not count the deaths of nursing home residents occurring in hospital as deaths in community-dwelling elderly. The correction was applied to one study (Hughes). |
| Sensitivity analyses | The seroreversion correction is calculated with a X^m^-fold correction to the IFR, not (1/X)^m^-fold. | Correction of typo in protocol. |
| Sensitivity analyses | Added clarification: The peak of the first epidemic wave was defined as one week before the date with the highest rolling average 7-day mortality (according to Worldometer, situational reports, or Wikipedia). The first epidemic wave was defined to end by the date with the lowest 7-day average of daily deaths since the beginning of the epidemic. If two or more dates were tied for peak values, we chose the date corresponding to the midpoint between the first and last one. | This definition had been missed in the protocol. |
| Sensitivity analyses | Added sensitivity analysis: excluding studies where the selected time point with highest seroprevalence was not the latest available (seroprevalence had declined in the latest timepoint). | Following comments arising during peer review |
| Data Synthesis and Analysis | We added a calculation of 95% CIs of IFRs based on extracted 95% CIs from seroprevalence estimates. Primarily, 95% confidence intervals are direct extractions from the seroprevalence studies. For studies that did not report such intervals, we complemented with a calculation using the number of sampled and seropositive elderly individuals (Clopper Pearson interval calculation).  For those that provided adjusted estimates for age brackets (e.g., 70-79, 80-89, and 90+), we combined estimates for each study using a fixed effects inverse variance meta-analysis (of arcsine transformed proportions) to obtain 95% CIs.  No further factors were introduced in the calculation beyond the adjustments made by seroprevalence study authors (except adjusting estimates for test performance using the Gladen-Rogan formula where applicable, see below). | In order to illustrate in Figure 1 the uncertainty of IFR estimates arising from seroprevalence sampling as reported by seroprevalence study authors, and to calculate the heterogeneity indicator I-squared.  The 95% CIs of IFRs do not take into account other sources of uncertainty than those adjusted by the seroprevalence authors, and should be interpreted as very conservative. |
| Calculated Data Variables | Added clarification: For studies that provided adjusted seroprevalence estimates for age brackets (e.g., 70-79, 80-89, and 90+) we calculated the number of infected individuals for each age bracket by multiplying with the corresponding population size. (For community-dwelling elderly, we first subtracted the nursing home population, assuming that the proportion of nursing home residents was equal across the age brackets). Then, we summarized the number of infected individuals in the age brackets to the number of infected individuals for (community-dwelling) elderly. | In order to be able to use adjusted seroprevalence estimates that were provided for age brackets that together formed an eligible elderly stratum. |
| Calculated Data Variables | For Qatar, and for the Dominican Republic, we could not retrieve information on the proportion of deaths accounted by nursing home residents, and we imputed a value of 10%. Imputation for these countries were not prespecified. | Qatar has a small number of nursing home residents relative to the population, although the number has increased in recent years. For the Dominican Republic, we imputed the same number prespecified for India. |
| Calculated Data Variables | We applied a non-prespecified correction for studies that excluded persons with diagnosed COVID-19 from participating in their sample, primarily using study authors’ corrections (e.g., PCR tests) or adding the number of identified COVID-19 cases in community-dwelling elderly for the location until the seroprevalence study midpoint.  For Gudbjartsson et al, we used the numbers based on seroprevalence and detection of ongoing infections.  For Kalish et al, we calculated the number of cases in community-dwelling elderly up to June 20 by retrieving the cumulative number of cases in elderly (≥65) from the COVID-19 Data Tracker at the US CDC (455,585) and subtracting the cumulative number of cases in nursing homes from KFF (240,138), resulting in 215,447 cases. This number was added to the number of infected community-dwelling elderly. | Added in order to represent correctly the number of infected persons. |
| Calculated Data Variables | Added calculation: Whenever no adjustment was made for test performance, we adjusted the estimates for test performance using the Gladen-Rogan formula (27). | Following comments arising during peer review |
| Calculated Data Variables | We did not adjust the main estimates for types of unmeasured antibodies (IgG/IgM/IgA). | Following comments arising during peer review |

^*^ Protocol, Open Science Framework: <https://osf.io/47cgb>

We are grateful to the editors and 7 peer-reviewers for all the constructive comments and for proposing additional analyses, as specified in the table above.

**Appendix Table 2**. Sources for COVID-19 mortality statistics and population statistics.

Note: Deaths in nursing home residents and population in nursing homes aim to capture all long-term care facilities (traditional nursing homes, assisted living, resident homes, other long-term care). For example, in the USA, the number of residents in federally regulated nursing homes is about 1.4 million, but more than 0.8 million additional people live in assisted-care living, resident homes, and other long-term care facilities. However, it is possible that only some types of long-term care facilities were included in the available data for different countries. If so, the number of deaths in long-term care facilities were under-estimated, deaths in community-dwelling populations were over-estimated and thus the estimated IFR in community dwelling populations may also be inflated accordingly. Moreover, documentation that death pertained to a long-term care resident was erratic in the early months for several countries and subsequent back-filing of missing historical information may not be complete. This may also result in under-estimation of long-term care deaths, thus over-estimation of deaths in community-dwelling population and therefore over-estimation also of IFR in community-dwelling populations. See more detailed discussion of these issues in https://covidtracking.com/analysis-updates/federal-covid-data-101-working-with-cms-nursing-home-data, https://covidtracking.com/analysis-updates/federal-covid-data-101-working-with-cms-nursing-home-data, and Comas-Herrera A, Zalakaín J, Litwin C, Hsu AT, Lemmon E, Henderson D and Fernández J-L Mortality associated with COVID-19 outbreaks in care homes: early international evidence. Article in LTCcovid.org, International Long-Term Care Policy Network, CPEC-LSE

| **Andorra (Study: Royo-Cebrecos)** |
| --- |
| Seroprevalence information: Seroprevalence study |
| Age distribution of COVID-19 deaths: Personal communication E. Heras Muxella and C. Royo-Cebrecos (i.e, obtained from the Head of the Ageing and Health Department in the Andorran Healthcare System) |
| Total number of deaths for the primary date: Personal communication E. Heras Muxella and C. Royo-Cebrecos (i.e, obtained from the Head of the Ageing and Health Department in the Andorran Healthcare System) |
| COVID-19 deaths in nursing home residents: Personal communication E. Heras Muxella and C. Royo-Cebrecos (i.e, obtained from the Head of the Ageing and Health Department in the Andorran Healthcare System) |
| Peak 7-day average of COVID-19 mortality: Worldometers.info |
| Population overall and in elderly:  https://www.estadistica.ad/serveiestudis/web/banc_dades4.asp?lang=4&codi_tema=2&codi_divisio=2783&codi_subtemes=8 |
| Population in nursing homes: Personal communication E. Heras Muxella and C. Royo-Cebrecos |
| Proportion of elderly among nursing home residents: Imputed (average of available values) |
| Comments:  Mortality peak is the midpoint of April 1, 2020, and April 16, 2020. |
|  |
| **Belgium (Study: Herzog)** |
| Seroprevalence information: Seroprevalence study and personal communication S. Herzog |
| Age distribution of COVID-19 deaths: Personal communication I. Peeters, Sciensano, the Belgian Institute for Health  (For younger age groups: Riffe, T., Acosta, E., the COVerAGE-DB team, Data Resource Profile: COVerAGE-DB: a global demographic database of COVID-19 cases and deaths, International Journal of Epidemiology, Volume 50, Issue 2, April 2021, Pages 390–390f, <https://doi.org/10.1093/ije/dyab027>. Accessed online as 10.17605/OSF.IO/MPWJQ.) |
| Total number of deaths for the primary date: Personal communication I. Peeters, Sciensano, the Belgian Institute for Health (number as of April 1, 2020) |
| COVID-19 deaths in nursing home residents: Situational report (COVID-19 – BULLETIN EPIDEMIOLOGIQUE  DU 10 AVRIL 2020, Sciensano) retrieved from https://covid-19.sciensano.be/fr/covid-19-situation-epidemiologique |
| Peak 7-day average of COVID-19 mortality: Worldometers.info |
| Population overall and in elderly (Jan 1, 2020): Dataset (Population de la Belgique par âge, au 1er janvier) retrieved from https://www.plan.be/databases/data-35-fr-perspectives_de_population_2020_2070 |
| Population in nursing homes: Personal communication I. Peeters, Sciensano, the Belgian Institute for Health |
| Proportion of elderly among nursing home residents: Imputed (average of available values) |
| Comments:  Only using Collection period 1 (Mar 30-Apr 5) while there are 4 more, ending July 4 (none of the others have >1000 elderly). |
|  |
| **Canada (Study: Saeed, Canadian Blood Services)** |
| Seroprevalence information: Seroprevalence study |
| Age distribution of COVID-19 deaths: Riffe, T., Acosta, E., the COVerAGE-DB team, Data Resource Profile: COVerAGE-DB: a global demographic database of COVID-19 cases and deaths, International Journal of Epidemiology, Volume 50, Issue 2, April 2021, Pages 390–390f, <https://doi.org/10.1093/ije/dyab027>. Accessed online as 10.17605/OSF.IO/MPWJQ. |
| Total number of deaths for the primary date: Riffe, T., Acosta, E., the COVerAGE-DB team, Data Resource Profile: COVerAGE-DB: a global demographic database of COVID-19 cases and deaths, International Journal of Epidemiology, Volume 50, Issue 2, April 2021, Pages 390–390f, <https://doi.org/10.1093/ije/dyab027>. Accessed online as 10.17605/OSF.IO/MPWJQ. |
| COVID-19 deaths in nursing home residents: Comas-Herrera A, Zalakaín J, Litwin C, Hsu AT, Lemmon E, Henderson D and Fernández J-L (2020) Mortality associated with COVID-19 outbreaks in care homes: early international evidence. Article in LTCcovid.org, International Long-Term Care Policy Network, CPEC-LSE, 26 June 2020. |
| Peak 7-day average of COVID-19 mortality: Worldometers.info |
| Population overall and elderly: StatCan Census 2016, dataset retrieved from https://www12.statcan.gc.ca/census-recensement/2016/dp-pd/index-eng.cfm |
| Population in nursing homes: StatCan Census 2016, dataset retrieved from https://www12.statcan.gc.ca/census-recensement/2016/dp-pd/index-eng.cfm |
| Proportion of elderly among nursing home residents: StatCan Census 2016, dataset retrieved from <https://www12.statcan.gc.ca/census-recensement/2016/dp-pd/index-eng.cfm> |
|  |
| **Canada [Ontario] (Study: Public Health Ontario)** |
| Seroprevalence information: Personal communication Public Health Ontario Communications |
| Age distribution of COVID-19 deaths: Enhanced Epidemiological Summary (COVID-19 Case Fatality, Case Identification, and Attack Rates in Ontario), May 17, retrieved from https://www.publichealthontario.ca/en/data-and-analysis/infectious-disease/covid-19-data-surveillance/weekly-epi-summary-archive |
| Total number of deaths for the primary date: Ontario COVID-19 database, Ontario Ministry of Health, “All Ontario: Case numbers and spread”, https://covid-19.ontario.ca/data/case-numbers-and-spread (accessed Jan 1, 2022) |
| COVID-19 deaths in nursing home residents: Dataset at https://data.ontario.ca/en/dataset/long-term-care-home-covid-19-data |
| Peak 7-day average of COVID-19 mortality: Dataset at https://covid-19.ontario.ca/data (accessed April 30, 2021) |
| Population overall and in elderly: StatCan Census 2016, dataset at https://www12.statcan.gc.ca/census-recensement/2016/dp-pd/index-eng.cfm |
| Population in nursing homes: StatCan Census 2016, dataset at https://www12.statcan.gc.ca/census-recensement/2016/dp-pd/index-eng.cfm |
| Proportion of elderly among nursing home residents: StatCan Census 2016, dataset at https://www12.statcan.gc.ca/census-recensement/2016/dp-pd/index-eng.cfm |
| Comments: 100% of the nursing home deaths reported were in elderly 70+. |
|  |
| **Canada [Alberta] (Study: Charlton)** |
| Seroprevalence information: Seroprevalence study |
| Age distribution of COVID-19 deaths: Riffe, T., Acosta, E., the COVerAGE-DB team, Data Resource Profile: COVerAGE-DB: a global demographic database of COVID-19 cases and deaths, International Journal of Epidemiology, Volume 50, Issue 2, April 2021, Pages 390–390f, <https://doi.org/10.1093/ije/dyab027>. Accessed online as 10.17605/OSF.IO/MPWJQ. |
| Total number of deaths for the primary date: Riffe, T., Acosta, E., the COVerAGE-DB team, Data Resource Profile: COVerAGE-DB: a global demographic database of COVID-19 cases and deaths, International Journal of Epidemiology, Volume 50, Issue 2, April 2021, Pages 390–390f, <https://doi.org/10.1093/ije/dyab027>. Accessed online as 10.17605/OSF.IO/MPWJQ. |
| COVID-19 deaths in nursing home residents: Canadian Institute for Health Information. The Impact of COVID-19 on Long-Term Care in Canada: Focus on the First 6 Months. Ottawa, ON: CIHI; 2021. |
| Peak 7-day average of COVID-19 mortality: COVID-19 Alberta Statistics. Summary data starting March 6, 2020. Government of Alberta. Retrieved from https://www.alberta.ca/stats/covid-19-alberta-statistics.htm#data-export |
| Population overall and in elderly: StatCan Census 2016, dataset at <https://www12.statcan.gc.ca/census-recensement/2016/dp-pd/index-eng.cfm> |
| Population in nursing homes: StatCan Census 2016, dataset at <https://www12.statcan.gc.ca/census-recensement/2016/dp-pd/index-eng.cfm> |
| Proportion of elderly among nursing home residents: StatCan Census 2016, dataset at https://www12.statcan.gc.ca/census-recensement/2016/dp-pd/index-eng.cfm |
| Comments: For COVID-19 deaths in nursing home residents, the number for Alberta, Canada, was unavailable historically. We therefore extrapolate from the Canada proportion of COVID-19 deaths in nursing home residents among all deaths (up to February 15, 2021): 67%, see reference listed above. |
|  |
| **Denmark [3 regions] (Study: Pedersen)** |
| Seroprevalence information: Seroprevalence study |
| Age distribution of COVID-19 deaths: Seroprevalence study |
| Total number of deaths for the primary date: Dataset at https://covid19.ssi.dk/overvagningsdata/download-fil-med-overvaagningdata [Deaths_over_time.csv] (Accessed February 17, 2021) |
| COVID-19 deaths in nursing home residents: Comas-Herrera A, Zalakaín J, Litwin C, Hsu AT, Lemmon E, Henderson D and Fernández J-L (2020) Mortality associated with COVID-19 outbreaks in care homes: early international evidence. Article in LTCcovid.org, International Long-Term Care Policy Network, CPEC-LSE, 26 June 2020. |
| Peak 7-day average of COVID-19 mortality: Worldometers.info |
| Population overall and in elderly: Seroprevalence study (based on Statistics Denmark) |
| Population in nursing homes: Dataset ("Antal beboere pa plejehjem") at https://sundhedsdatastyrelsen.dk/da/tal-og-analyser/analyser-og-rapporter/sundhedsvaesenet/plejehjem |
| Proportion of elderly among nursing home residents: Statistics Denmark, dataset (“Indskrevne i pleje- og ældreboliger efter område, tid, alder og foranstaltningsart”) |
| Comments:  Proportion of those aged ≥85 years among all elderly (≥70 years) is derived from Statistics Denmark (the whole country): 14.86% |
|  |
| **Denmark (Study: Espenhain)** |
| Seroprevalence information: Seroprevalence study |
| Age distribution of COVID-19 deaths: Riffe, T., Acosta, E., the COVerAGE-DB team, Data Resource Profile: COVerAGE-DB: a global demographic database of COVID-19 cases and deaths, International Journal of Epidemiology, Volume 50, Issue 2, April 2021, Pages 390–390f, <https://doi.org/10.1093/ije/dyab027>. Accessed online as 10.17605/OSF.IO/MPWJQ. |
| Total number of deaths for the primary date: Riffe, T., Acosta, E., the COVerAGE-DB team, Data Resource Profile: COVerAGE-DB: a global demographic database of COVID-19 cases and deaths, International Journal of Epidemiology, Volume 50, Issue 2, April 2021, Pages 390–390f, <https://doi.org/10.1093/ije/dyab027>. Accessed online as 10.17605/OSF.IO/MPWJQ. |
| COVID-19 deaths in nursing home residents: Comas-Herrera A, Zalakaín J, Litwin C, Hsu AT, Lemmon E, Henderson D and Fernández J-L (2020) Mortality associated with COVID-19 outbreaks in care homes: early international evidence. Article in LTCcovid.org, International Long-Term Care Policy Network, CPEC-LSE, 26 June 2020. |
| Peak 7-day average of COVID-19 mortality: Worldometers.info |
| Population overall and in elderly: Dataset at https://www.statistikbanken.dk/statbank5a/selectvarval/saveselections.asp |
| Population in nursing homes: Dataset ("Antal beboere pa plejehjem") at https://sundhedsdatastyrelsen.dk/da/tal-og-analyser/analyser-og-rapporter/sundhedsvaesenet/plejehjem |
| Proportion of elderly among nursing home residents: Statistics Denmark, dataset (“Indskrevne i pleje- og ældreboliger efter område, tid, alder og foranstaltningsart”) |
| Comments: |
|  |
| **Dominican Republic (Study: Paulino-Ramirez)** |
| Seroprevalence information: Seroprevalence study |
| Age distribution of COVID-19 deaths: Boletín especial #65, Enfermedad por Coronavirus 2019 (COVID-19), Figura 4, situational report with data from May 22, retrieved from https://coronavirusrd.gob.do/documentos/boletines/ |
| Total number of deaths for the primary date: Boletín especial #65, Enfermedad por Coronavirus 2019 (COVID-19), situational report with data from May 22, retrieved from https://coronavirusrd.gob.do/documentos/boletines/ |
| COVID-19 deaths in nursing home residents: Unavailable, imputed |
| Peak 7-day average of COVID-19 mortality: Worldometers.info |
| Population overall and in elderly: populationpyramid.net |
| Population in nursing homes: ”Living Arrangements of Older Persons: A Report on an Expanded International Dataset”, United Nations (2017), Table A.III.1, retrieved from https://www.un.org/en/development/desa/population/publications/pdf/ageing/LivingArrangements.pdf |
| Proportion of elderly among nursing home residents: Imputed (average of available values) |
|  |
| **France, Ile-de-France (Study: Carrat)** |
| Seroprevalence information: Seroprevalence study |
| Age distribution of COVID-19 deaths: (1) DONNÉES DE SURVEILLANCE. SURVEILLANCE DE LA MORTALITÉ AU COURS DE L'ÉPIDÉMIE DE COVID-19 DU 2 MARS AU 31 MAI 2020 EN FRANCE, Santé publique France; (2) Point épidémio régional Ile-de-France Spécial COVID-19 28 mai 2020, Santé publique France |
| Total number of deaths for the primary date: DONNÉES DE SURVEILLANCE. SURVEILLANCE DE LA MORTALITÉ AU COURS DE L'ÉPIDÉMIE DE COVID-19 DU 2 MARS AU 31 MAI 2020 EN FRANCE, Santé publique France |
| COVID-19 deaths in nursing home residents: DONNÉES DE SURVEILLANCE. SURVEILLANCE DE LA MORTALITÉ AU COURS DE L'ÉPIDÉMIE DE COVID-19 DU 2 MARS AU 31 MAI 2020 EN FRANCE, Santé publique France |
| Peak 7-day average of COVID-19 mortality: DONNÉES DE SURVEILLANCE. SURVEILLANCE DE LA MORTALITÉ AU COURS DE L'ÉPIDÉMIE DE COVID-19 DU 2 MARS AU 31 MAI 2020 EN FRANCE, Santé publique France |
| Population overall: Webpage https://www.insee.fr/en/statistiques/3696316?p1=r11&p2=r97&lang=en&annee=2020 (accessed May 31, 2021) |
| Population in elderly: Webpage https://www.insee.fr/en/statistiques/3696316?p1=r11&p2=r97&lang=en&annee=2020 (accessed May 31, 2021) |
| Population in nursing homes: Webpage https://www.insee.fr/fr/statistiques/4124769#tableau-figure4 (accessed May 31, 2021) |
| Proportion of elderly among nursing home residents: Imputed (average of available values) |
| Comments:  The report “Surveillance de la mortalité …” by Santé publique France, Table 5, lists the number of COVID-19 deaths in hospitalized patients and the nursing home residents’ COVID-19 deaths by May 31, per region. In Ile-de-France: 7262 hospitalized, 4368 EHPAD/EMS (total 7262+4368).  The region of Ile-de-France has a situational report from May 28, listing age distribution of deaths occurring in hospitalized patients up to May 26 in Tableau 5: 5229/6930 (age cutoff 70 years) with age information. Total number of deaths were 6958. Note that this number does not include the nursing home residents’ deaths, which is here added using the proportion of deaths in the Santé publique report, May 31.  Total number of deaths = (4368/11630)*6958+6958 = 9571. Number of deaths in elderly ≥70 years = (4368/11630)*6958*0.95+5229 = 7712.  Three regions were sampled in the study by Carrat, and two were eligible (the ones with >1000 participants 70+): Ile-de-France and Nouvelle Aquitaine. Using primary outcome (ELISA-S). Figure 2 (weighted estimates) and Suppl figure 5 (unweighted). |
|  |
| **France, Nouvelle Aquitaine (Study: Carrat)** |
| Seroprevalence information: Seroprevalence study |
| Age distribution of COVID-19 deaths: Point épidémio régional Nouvelle-Aquitaine Spécial COVID-19 28 mai 2020, Santé publique France |
| Total number of deaths for the primary date: Point épidémio régional Nouvelle-Aquitaine Spécial COVID-19 28 mai 2020, Santé publique France |
| COVID-19 deaths in nursing home residents: DONNÉES DE SURVEILLANCE. SURVEILLANCE DE LA MORTALITÉ AU COURS DE L'ÉPIDÉMIE DE COVID-19 DU 2 MARS AU 31 MAI 2020 EN FRANCE, Santé publique France |
| Peak 7-day average of COVID-19 mortality: DONNÉES DE SURVEILLANCE. SURVEILLANCE DE LA MORTALITÉ AU COURS DE L'ÉPIDÉMIE DE COVID-19 DU 2 MARS AU 31 MAI 2020 EN FRANCE, Santé publique France |
| Population overall: Webpage https://www.insee.fr/en/statistiques/3696316?p1=r11&p2=r97&lang=en&annee=2020 (accessed May 31, 2021) |
| Population in elderly: Webpage https://www.insee.fr/en/statistiques/3696316?p1=r11&p2=r97&lang=en&annee=2020 (accessed May 31, 2021) |
| Population in nursing homes: Webpage https://www.insee.fr/fr/statistiques/4294178#tableau-figure2 (accessed May 31, 2021) |
| Proportion of elderly among nursing home residents: Imputed (average of available values) |
| Comments:  The report “Surveillance de la mortalité …” by Santé publique France, Table 5, lists the number of COVID-19 deaths in hospitalized patients and the nursing home residents’ COVID-19 deaths, per region. Nouvelle Aquitaine: 406 hospitalized, 129 EHPAD/EMS (total 406+129).  The region of Nouvelle Aquitaine has a situational report from May 28, listing age distribution of all deaths up to May 25 in Tableau 7: 409/448 (age cutoff 65 years).  Population in nursing homes: Derived from the Insee webpage Figure 2, Number of dependent seniors (2016), n=331,200. In text: "in 2016, 8 out of 10 dependent elderly people lived at home" [and the others in institutions].  Three regions were sampled in the study by Carrat, and two were eligible (the ones with >1000 participants 70+): Ile-de-France and Nouvelle Aquitaine. Using primary outcome (ELISA-S). Figure 2 (weighted estimates) and Suppl figure 5 (unweighted). |
|  |
| **France (Study: Warszawski / INSERM)** |
| Seroprevalence information: Seroprevalence study |
| Age distribution of COVID-19 deaths: Point épidémiologique hebdomadaire du 03 décembre 2020, Santé publique France, Tableau 2 and Tableau 4, retrieved from https://www.santepubliquefrance.fr/recherche/#search=COVID%2019%20%20%20point%20epidemiologique&publications=donn%C3%A9es&regions=National&sort=date |
| Total number of deaths for the primary date: Point épidémiologique hebdomadaire du 03 décembre 2020, Santé publique France, Tableau 2 and Tableau 4, retrieved from https://www.santepubliquefrance.fr/recherche/#search=COVID%2019%20%20%20point%20epidemiologique&publications=donn%C3%A9es&regions=National&sort=date |
| COVID-19 deaths in nursing home residents: Point épidémiologique hebdomadaire du 03 décembre 2020, Santé publique France, Tableau 2 and Tableau 4, retrieved from https://www.santepubliquefrance.fr/recherche/#search=COVID%2019%20%20%20point%20epidemiologique&publications=donn%C3%A9es&regions=National&sort=date |
| Peak 7-day average of COVID-19 mortality: Worldometers.info |
| Population overall and in elderly: INSEE (Age structure of the population on 1^st^ January 2020, metropolitan France), dataset at https://www.insee.fr/en/statistiques/5015921?sommaire=5015923 |
| Population in nursing homes: INSEE (Structures d'hébergement pour personnes âgées, December 31, 2016), dataset at https://www.insee.fr/fr/statistiques/3676717?sommaire=3696937 |
| Proportion of elderly among nursing home residents: Imputed (average of available values) |
| Comments:  The number of deaths in elderly is derived from Tableau 4 (deaths occurring in hospital) and Tableau 2 (deaths in care homes) assuming that 98% of the 16,664 deaths in care homes were among persons 65 years and older according to our prespecification. |
|  |
| **Hungary (Study: Merkely)** |
| Seroprevalence information: Personal communication Z. Vokó |
| Age distribution of COVID-19 deaths: Early phase of the COVID-19 outbreak in Hungary and post-lockdown scenarios  Gergely Röst, Ferenc A. Bartha, Norbert Bogya, Péter Boldog, Attila Dénes, Tamás Ferenci, Krisztina J. Horváth, Attila Juhász, Csilla Nagy, Tamás Tekeli, Zsolt Vizi, Beatrix Oroszi. (Version 1) medRxiv 2020.06.02.20119313; doi: https://doi.org/10.1101/2020.06.02.20119313 |
| Total number of deaths for the primary date: Worldometers.info (accessed June 1, 2021) |
| COVID-19 deaths in nursing home residents: Comas-Herrera A, Zalakaín J, Litwin C, Hsu AT, Lane N and Fernández J-L (2020) Mortality associated with COVID-19 outbreaks in care homes: early international evidence. Article in LTCcovid.org, International Long-Term Care Policy Network, CPEC-LSE, 21 May 2020. |
| Peak 7-day average of COVID-19 mortality: Worldometers.info |
| Population overall and in elderly: populationpyramid.net |
| Population in nursing homes: Comas-Herrera A, Zalakaín J, Lemmon E, Henderson D, Litwin C, Hsu AT, Schmidt AE, Arling G and Fernández J-L (2020) Mortality associated with COVID-19 in care homes: international evidence. Article in LTCcovid.org, International Long-Term Care Policy Network, CPEC-LSE, 14 October. (page 10, in text) |
| Proportion of elderly among nursing home residents: Comas-Herrera A, Zalakaín J, Lemmon E, Henderson D, Litwin C, Hsu AT, Schmidt AE, Arling G and Fernández J-L (2020) Mortality associated with COVID-19 in care homes: international evidence. Article in LTCcovid.org, International Long-Term Care Policy Network, CPEC-LSE, 14 October. (page 10, in text) |
|  |
| **Iceland (Study: Gudbjartsson)** |
| Seroprevalence information: Seroprevalence study and https://www.ruv.is/frett/2020/04/20/annad-andlat-ur-covid-19-a-hjukrunarheimilinu-bergi (5 infected nursing home residents) |
| Age distribution of COVID-19 deaths: Webpage, https://www.covid.is/data-old (last updated: June 14, 2020; accessed: May 29, 2021) |
| Total number of deaths for the primary date: Webpage, https://www.covid.is/data-old (last updated: June 14, 2020; accessed: May 29, 2021) |
| COVID-19 deaths in nursing home residents: Wikipedia, https://en.wikipedia.org/wiki/COVID-19_pandemic_in_Iceland (accessed: May 29, 2021) |
| Peak 7-day average of COVID-19 mortality: Worldometers.info |
| Population overall and in elderly: populationpyramid.net |
| Population in nursing homes: Dataset "Occupants of retirement homes and nursing homes and wards by type of institution 1993-2010" (selection: 2010, In nursing homes) retrieved from https://px.hagstofa.is/pxen/pxweb/en/Samfelag/Samfelag__felagsmal__aldradir/HEI03004.px |
| Proportion of elderly among nursing home residents: Imputed (average of available values) |
| Comments:  The seroprevalence study excluded previously COVID-19-diagnosed persons, and presents prevalence based on antibody tests and PCR tests. The estimate given is the number of infected Icelanders of age 70+ (i.e., the absolute number, not seroprevalence), Table S7 (n=165.4). In the calculations to follow, we subtract 5 infections in nursing homes, see website source. Gudbjartsson’s estimated IFR is 4.4% (1.9%, 8.3%). Since no confidence intervals are given for the number of infected persons (or seroprevalence), their confidence intervals for IFR are used in Figure 1.  Mortality peak date is the midpoint between March 27 and April 23. |
|  |
| **India (Study: Murhekar)** |
| Seroprevalence information: Seroprevalence study |
| Age distribution of COVID-19 deaths: Press conference by India's health secretary October 13, referenced at https://science.thewire.in/health/india-covid-19-mortality-comorbidities-age-health-ministry/ |
| Total number of deaths for the primary date: Worldometers.info (accessed June 1, 2021) |
| COVID-19 deaths in nursing home residents: Unavailable, imputed |
| Peak 7-day average of COVID-19 mortality: Worldometers.info |
| Population overall and in elderly: populationpyramid.net |
| Population in nursing homes: “Report on old age facilities in India”, Tata Trusts, Samarth, and United Nations Population Fund (2018), retrieved from https://www.tatatrusts.org/upload/pdf/report-on-old-age-facilities-in-india.pdf |
|  |
| **India [Tamil Nadu] (Study: Malani)** |
| Seroprevalence information: Seroprevalence study |
| Age distribution of COVID-19 deaths: Seroprevalence study |
| Total number of deaths for the primary date: Seroprevalence study |
| COVID-19 deaths in nursing home residents: Unavailable, imputed |
| Peak 7-day average of COVID-19 mortality: Wikipedia |
| Population overall and in elderly: Seroprevalence study (Census 2011) |
| Population in nursing homes: “Report on old age facilities in India”, Tata Trusts, Samarth, and United Nations Population Fund (2018), retrieved from https://www.tatatrusts.org/upload/pdf/report-on-old-age-facilities-in-india.pdf (proportion in India applied to population in Tamil Nadu) |
| Proportion of elderly among nursing home residents: Imputed (average of available values) |
| Comments:  Mortality peak is the midpoint of Sept 15, 2020, and Sept 19, 2020. |
|  |
| **Israel (Study: Reicher)** |
| Seroprevalence information: Seroprevalence study |
| Age distribution of COVID-19 deaths: Riffe, T., Acosta, E., the COVerAGE-DB team, Data Resource Profile: COVerAGE-DB: a global demographic database of COVID-19 cases and deaths, International Journal of Epidemiology, Volume 50, Issue 2, April 2021, Pages 390–390f, https://doi.org/10.1093/ije/dyab027. Accessed online as 10.17605/OSF.IO/MPWJQ. |
| Total number of deaths for the primary date: Worldometers.info |
| COVID-19 deaths in nursing home residents: Comas-Herrera A, Zalakaín J, Litwin C, Hsu AT, Lemmon E, Henderson D and Fernández J-L (2020) Mortality associated with COVID-19 outbreaks in care homes: early international evidence. Article in LTCcovid.org, International Long-Term Care Policy Network, CPEC-LSE, 26 June 2020 |
| Peak 7-day average of COVID-19 mortality: Worldometers.info |
| Population overall and in elderly: populationpyramid.net |
| Population in nursing homes: Muhsen K, Maimon N, Mizrahi A, et al. Effects of BNT162b2 Covid-19 vaccine booster in long-term care facilities in Israel. N Engl J Med. DOI: 10.1056/NEJMc2117385 |
| Proportion of elderly among nursing home residents: Imputed (average of available values) |
| Comments: Number of long-term beds in Israel (2020): 17.7/1000 persons aged 65 years or older. |
|  |
| **Italy (Study: Istat)** |
| Seroprevalence information: Seroprevalence study |
| Age distribution of COVID-19 deaths: Epidemia COVID-19, Aggiornamento nazionale, 23 giugno 2020 – ore 11:00, DATA PUBBLICAZIONE: 26 GIUGNO 2020, ISS, retrieved from https://www.epicentro.iss.it/coronavirus/bollettino/Bollettino-sorveglianza-integrata-COVID-19_23-giugno-2020.pdf |
| Total number of deaths for the primary date: Worldometers.info |
| COVID-19 deaths in nursing home residents: (1) Comas-Herrera A, Zalakaín J, Litwin C, Hsu AT, Lemmon E, Henderson D and Fernández J-L (2020) Mortality associated with COVID-19 outbreaks in care homes: early international evidence. Article in LTCcovid.org, International Long-Term Care Policy Network, CPEC-LSE, 26 June 2020 [for the absolute number of nursing home residents estimated to have died by May 5, 2020, n=9212]; (2) Worldometers.info [for the total number of deaths at the corresponding date, n=29389] |
| Peak 7-day average of COVID-19 mortality: Worldometers.info |
| Population overall and in elderly: populationpyramid.net |
| Population in nursing homes: Comas-Herrera A, Zalakaín J, Litwin C, Hsu AT, Lemmon E, Henderson D and Fernández J-L (2020) Mortality associated with COVID-19 outbreaks in care homes: early international evidence. Article in LTCcovid.org, International Long-Term Care Policy Network, CPEC-LSE, 26 June 2020 |
| Proportion of elderly among nursing home residents: Imputed (average of available values) |
| Comments:  COVID-19 deaths in nursing home residents, calculated from LTC Policy Network report June 26, Table 2: 3.1% of 297,158 residents were estimated to have died of COVID-19 at that point. |
|  |
| **Netherlands (Study: Vos)** |
| Seroprevalence information: Seroprevalence study |
| Age distribution of COVID-19 deaths: Epidemiologische situatie COVID-19 in Nederland. Rijksinstituut voor Volksgezondheid en Milieu – RIVM. 21 juni 2020, 10:00. Retrieved from: https://www.rivm.nl/coronavirus-covid-19/actueel/wekelijkse-update-epidemiologische-situatie-covid-19-in-nederland |
| Total number of deaths for the primary date: Epidemiologische situatie COVID-19 in Nederland. Rijksinstituut voor Volksgezondheid en Milieu – RIVM. 21 juni 2020, 10:00. Retrieved from: https://www.rivm.nl/coronavirus-covid-19/actueel/wekelijkse-update-epidemiologische-situatie-covid-19-in-nederland |
| COVID-19 deaths in nursing home residents: Rijksinstituut voor Volksgezondheid en Milieu (RIVM) “open data set on nursing home care”, retrieved 2021-12-17 from https://coronadashboard.government.nl/verantwoording#nursing-homes |
| Peak 7-day average of COVID-19 mortality: Worldometers.info |
| Population overall and in elderly: Statistics Netherlands (CBS), Population pyramid: Age composition in the Nederlands 2021. Retrieved from: https://www.cbs.nl/en-gb/visualisations/dashboard-population/population-pyramid |
| Population in nursing homes: Statistics Netherlands (CBS), Households; size, composition, position in the household, 1 January (2021). Retrieved from: https://www.cbs.nl/en-gb/society/population |
| Proportion of elderly among nursing home residents: Imputed (average of available values) |
| Comments: |
|  |
| **Qatar (Study: Abu-Raddad)** |
| Seroprevalence information: Seroprevalence study |
| Age distribution of COVID-19 deaths: Seroprevalence study |
| Total number of deaths for the primary date: Worldometers.info (accessed June 1, 2021) |
| COVID-19 deaths in nursing home residents: Unavailable, imputed |
| Peak 7-day average of COVID-19 mortality: Worldometers.info |
| Population overall and in elderly: populationpyramid.net |
| Population in nursing homes: ”Living Arrangements of Older Persons: A Report on an Expanded International Dataset”, United Nations (2017), Table A.III.1, retrieved from https://www.un.org/en/development/desa/population/publications/pdf/ageing/LivingArrangements.pdf |
| Proportion of elderly among nursing home residents: Imputed (average of available values) |
| Comments:  Confidence intervals for adjusted seroprevalence not reported in study, and so calculated using the inferred adjusted number of seropositive individuals.  Population in nursing homes: The number for females (0.5% of elderly living in institutions) selected since the definition of institutional living included other collective housing than elderly homes, and the male population includes a large number of workers living in collective quarters.  Mortality peak date is the midpoint between June 21, 2020, and July 10, 2020. |
|  |
| **Spain (Study: Ministerio de Sanidad and Instituto de Salud Carlos III)** |
| Seroprevalence information: Seroprevalence study |
| Age distribution of COVID-19 deaths: Dataset "casos_hosp_uci_def_sexo_edad_provres.csv" retrieved from https://cnecovid.isciii.es/covid19/#documentación-y-datos, accessed 2021-06-23 |
| Total number of deaths for the primary date: Dataset "casos_hosp_uci_def_sexo_edad_provres.csv" retrieved from https://cnecovid.isciii.es/covid19/#documentación-y-datos, accessed 2021-06-23 |
| COVID-19 deaths in nursing home residents: Comas-Herrera A, Zalakaín J, Lemmon E, Henderson D, Litwin C, Hsu AT, Schmidt AE, Arling G, Kruse F and Fernández J-L (2020) Mortality associated with COVID-19 in care homes: international evidence. Article in LTCcovid.org, International Long-Term Care Policy Network, CPEC-LSE, 1st February 2021. |
| Peak 7-day average of COVID-19 mortality: Worldometers.info |
| Population overall and in elderly: populationpyramid.net |
| Population in nursing homes: Webpage, http://envejecimientoenred.es/una-nueva-estimacion-de-poblacion-en-residencias-de-mayores/ (July 20, 2020; accessed May 29, 2021) |
| Proportion of elderly among nursing home residents: Webpage, http://envejecimientoenred.es/una-nueva-estimacion-de-poblacion-en-residencias-de-mayores/ (July 20, 2020; accessed May 29, 2021) |
|  |
| **United Kingdom (Study: UK Biobank)** |
| Seroprevalence information: Seroprevalence study |
| Age distribution of COVID-19 deaths: Deaths registered weekly in England and Wales, provisional, Office of National Statistics, dataset (sheet "UK - Covid-19 - Weekly reg") retrieved from https://www.ons.gov.uk/peoplepopulationandcommunity/birthsdeathsandmarriages/deaths/datasets/weeklyprovisionalfiguresondeathsregisteredinenglandandwales (accessed February 19, 2021). Note that this dataset includes numbers for the United Kingdom, not only England and Wales. |
| Total number of deaths for the primary date: Deaths registered weekly in England and Wales, provisional, Office of National Statistics, dataset (sheet "UK - Covid-19 - Weekly reg") retrieved from https://www.ons.gov.uk/peoplepopulationandcommunity/birthsdeathsandmarriages/deaths/datasets/weeklyprovisionalfiguresondeathsregisteredinenglandandwales (accessed February 19, 2021). Note that this dataset includes numbers for the United Kingdom, not only England and Wales. |
| COVID-19 deaths in nursing home residents: Comas-Herrera A, Zalakaín J, Lemmon E, Henderson D, Litwin C, Hsu AT, Schmidt AE, Arling G and Fernández J-L (2020) Mortality associated with COVID-19 in care homes: international evidence. Article in LTCcovid.org, International Long-Term Care Policy Network, CPEC-LSE, 14 October |
| Peak 7-day average of COVID-19 mortality: Worldometers.info |
| Population overall and in elderly: Population estimates for the UK, England and Wales, Scotland and Northern Ireland: mid-2019, using April 2020 local authority district codes, Office for National Statistics (sheet “MYE1”), retrieved from https://www.ons.gov.uk/peoplepopulationandcommunity/populationandmigration/populationestimates/datasets/populationestimatesforukenglandandwalesscotlandandnorthernireland, "Mid-2019: April 2020 local authority district codes" |
| Population in nursing homes: Comas-Herrera A, Zalakaín J, Lemmon E, Henderson D, Litwin C, Hsu AT, Schmidt AE, Arling G and Fernández J-L (2020) Mortality associated with COVID-19 in care homes: international evidence. Article in LTCcovid.org, International Long-Term Care Policy Network, CPEC-LSE, 14 October (Table 2, column “% of pop living in care homes”) |
| Proportion of elderly among nursing home residents: Bowman et al 2004, Age and Ageing 2004; 33: 561–566, doi:10.1093/ageing/afh177 |
| Comments:  The alternative sampling period May 27, 2020, to July 6, 2020, is discarded according to eligibility criteria (lower seroprevalence). |
|  |
| **United Kingdom [England] (Study: Ward)** |
| Seroprevalence information: Seroprevalence study |
| Age distribution of COVID-19 deaths: Deaths registered weekly in England and Wales, provisional, Office of National Statistics, dataset (sheet "UK - Covid-19 - Weekly occurrences") retrieved from https://www.ons.gov.uk/peoplepopulationandcommunity/birthsdeathsandmarriages/deaths/datasets/weeklyprovisionalfiguresondeathsregisteredinenglandandwales (accessed February 19, 2021). The age distribution for England and Wales is used. |
| Total number of deaths for the primary date: Deaths registered weekly in England and Wales, provisional, Office of National Statistics, dataset (sheet "UK - Covid-19 - Weekly occurrences") retrieved from https://www.ons.gov.uk/peoplepopulationandcommunity/birthsdeathsandmarriages/deaths/datasets/weeklyprovisionalfiguresondeathsregisteredinenglandandwales (accessed February 19, 2021). |
| COVID-19 deaths in nursing home residents: Dataset ("julydeathsinvolvingcovid19inthecaresectordataset02072020155122.xlsx", sheet: “Table 1”), retrieved from https://www.ons.gov.uk/peoplepopulationandcommunity/birthsdeathsandmarriages/deaths/datasets/deathsinvolvingcovid19inthecaresectorenglandandwales (accessed February 19, 2021) |
| Peak 7-day average of COVID-19 mortality: Deaths registered weekly in England and Wales, provisional, Office of National Statistics, dataset (sheet "UK - Covid-19 - Daily occurrences") retrieved from https://www.ons.gov.uk/peoplepopulationandcommunity/birthsdeathsandmarriages/deaths/datasets/weeklyprovisionalfiguresondeathsregisteredinenglandandwales (accessed February 19, 2021). |
| Population overall and in elderly: Population estimates for the UK, England and Wales, Scotland and Northern Ireland: mid-2019, using April 2020 local authority district codes, Office for National Statistics (sheet “MYE1”), retrieved from https://www.ons.gov.uk/peoplepopulationandcommunity/populationandmigration/populationestimates/datasets/populationestimatesforukenglandandwalesscotlandandnorthernireland, "Mid-2019: April 2020 local authority district codes" |
| Population in nursing homes: Comas-Herrera A, Zalakaín J, Lemmon E, Henderson D, Litwin C, Hsu AT, Schmidt AE, Arling G and Fernández J-L (2020) Mortality associated with COVID-19 in care homes: international evidence. Article in LTCcovid.org, International Long-Term Care Policy Network, CPEC-LSE, 14 October (Table 2, column “% of pop living in care homes”) |
| Proportion of elderly among nursing home residents: Dataset at http://www.nomisweb.co.uk/census/2011/all_tables?release=3.4, "DC4210EWIa", England |
| Comments: The age distribution of COVID-19 mortality for England and Wales is used. |
|  |
| **United Kingdom [England and Wales] (Study: Public Health England)** |
| Seroprevalence information: Seroprevalence study |
| Age distribution of COVID-19 deaths: Deaths registered weekly in England and Wales, provisional, Office of National Statistics, dataset (sheet "UK - Covid-19 - Weekly occurrences") retrieved from https://www.ons.gov.uk/peoplepopulationandcommunity/birthsdeathsandmarriages/deaths/datasets/weeklyprovisionalfiguresondeathsregisteredinenglandandwales (accessed February 19, 2021). |
| Total number of deaths for the primary date: Deaths registered weekly in England and Wales, provisional, Office of National Statistics, dataset (sheet "UK - Covid-19 - Weekly occurrences") retrieved from https://www.ons.gov.uk/peoplepopulationandcommunity/birthsdeathsandmarriages/deaths/datasets/weeklyprovisionalfiguresondeathsregisteredinenglandandwales (accessed February 19, 2021). |
| COVID-19 deaths in nursing home residents: Dataset ("julydeathsinvolvingcovid19inthecaresectordataset02072020155122.xlsx", sheet: “Table 1”), retrieved from https://www.ons.gov.uk/peoplepopulationandcommunity/birthsdeathsandmarriages/deaths/datasets/deathsinvolvingcovid19inthecaresectorenglandandwales (accessed February 19, 2021) |
| Peak 7-day average of COVID-19 mortality: Deaths registered weekly in England and Wales, provisional, Office of National Statistics, dataset (sheet "UK - Covid-19 - Daily occurrences") retrieved from https://www.ons.gov.uk/peoplepopulationandcommunity/birthsdeathsandmarriages/deaths/datasets/weeklyprovisionalfiguresondeathsregisteredinenglandandwales (accessed February 19, 2021). |
| Population overall and in elderly: Population estimates for the UK, England and Wales, Scotland and Northern Ireland: mid-2019, using April 2020 local authority district codes, Office for National Statistics (sheet “MYE1”), retrieved from https://www.ons.gov.uk/peoplepopulationandcommunity/populationandmigration/populationestimates/datasets/populationestimatesforukenglandandwalesscotlandandnorthernireland, "Mid-2019: April 2020 local authority district codes" |
| Population in nursing homes: Comas-Herrera A, Zalakaín J, Lemmon E, Henderson D, Litwin C, Hsu AT, Schmidt AE, Arling G and Fernández J-L (2020) Mortality associated with COVID-19 in care homes: international evidence. Article in LTCcovid.org, International Long-Term Care Policy Network, CPEC-LSE, 14 October (Table 2, column “% of pop living in care homes”, England) |
| Proportion of elderly among nursing home residents: Dataset at http://www.nomisweb.co.uk/census/2011/all_tables?release=3.4, "DC4210EWIa", England and Wales |
| Comments:  First of two sampling periods, chosen according to eligibility criteria (highest seroprevalence). |
|  |
| **United Kingdom [Greater Glasgow and Clyde, Scotland] (Study: Hughes)** |
| Seroprevalence information: Seroprevalence study |
| Age distribution of COVID-19 deaths: Weekly deaths by health board, age group, sex and cause, 2020 and 2021 , dataset retrieved from https://www.nrscotland.gov.uk/files/statistics/covid19/weekly-deaths-by-sex-age-group-health-board-2020-2021.xlsx (accessed February 19, 2021; date range specified according to primary date) |
| Total number of deaths for the primary date: Weekly deaths by health board, age group, sex and cause, 2020 and 2021, dataset retrieved from https://www.nrscotland.gov.uk/files/statistics/covid19/weekly-deaths-by-sex-age-group-health-board-2020-2021.xlsx (accessed February 19, 2021; date range specified according to primary date) |
| COVID-19 deaths in nursing home residents: Weekly deaths by area and location, 2020 and 2021 (sheet: “HB COVID”), dataset retrieved from https://www.nrscotland.gov.uk/files/statistics/covid19/weekly-deaths-by-location-health-board-council-area-2020-2021.xlsx |
| Peak 7-day average of COVID-19 mortality: Deaths involving coronavirus (COVID-19) in Scotland (Table 1 (2020), dataset retrieved from https://www.nrscotland.gov.uk/statistics-and-data/statistics/statistics-by-theme/vital-events/general-publications/weekly-and-monthly-data-on-births-and-deaths/deaths-involving-coronavirus-covid-19-in-scotland |
| Population overall and in elderly: "Mid-2019 population estimates Scotland" (sheet: “Table 3”), dataset retrieved from https://www.nrscotland.gov.uk/statistics-and-data/statistics/statistics-by-theme/population/population-estimates/mid-year-population-estimates/mid-2019 |
| Population in nursing homes: Scottish Care Home Census, Public Health Scotland (PHS), "Care Home Census Data Tables", (sheet: “Table 3”), dataset retrieved from https://beta.isdscotland.org/find-publications-and-data/health-and-social-care/social-and-community-care/care-home-census-for-adults-in-scotland/ |
| Proportion of elderly among nursing home residents: Scottish Care Home Census, Public Health Scotland (PHS), "Care Home Census Data Tables", (sheet: “Table 8”), dataset retrieved from https://beta.isdscotland.org/find-publications-and-data/health-and-social-care/social-and-community-care/care-home-census-for-adults-in-scotland/ |
| Comments:  NHSGGC (base for seroprevalence study sampling) covers the following local authorities: Inverclyde, Renfrewshire, East Renfrewshire, Glasgow City, East Dunbartonshire and West Dunbartonshire.  COVID-19 death statistics for nursing home residents do not include deaths occurring in hospital, and so was corrected with a factor of 1.225 (the median of the ratio of deaths in nursing home residents / deaths occurring in nursing homes, in the European countries with such data in the Long-term Care Policy Network report October 14, Comas-Herrera et al). |
|  |
| **United States of America (Study: Anand)** |
| Seroprevalence information: Seroprevalence study |
| Age distribution of COVID-19 deaths: Dataset retrieved from https://data.cdc.gov/NCHS/Provisional-COVID-19-Death-Counts-by-Sex-Age-and-W/vsak-wrfu (accessed February 19, 2021) |
| Total number of deaths for the primary date: Worldometers.info (accessed June 1, 2021) |
| COVID-19 deaths in nursing home residents: Kaiser Family Foundation (KFF), https://www.kff.org/policy-watch/this-week-in-coronavirus-july-10-to-july-16/ (accessed May 5, 2021) |
| Peak 7-day average of COVID-19 mortality: Worldometers.info |
| Population overall and in elderly: "Annual Estimates of the Resident Population for Selected Age Groups by Sex: April 1, 2010 to July 1, 2019", dataset retrieved from https://www.census.gov/data/tables/time-series/demo/popest/2010s-national-detail.html |
| Population in nursing homes: Comas-Herrera A, Zalakaín J, Lemmon E, Henderson D, Litwin C, Hsu AT, Schmidt AE, Arling G and Fernández J-L (2020) Mortality associated with COVID-19 in care homes: international evidence. Article in LTCcovid.org, International Long-Term Care Policy Network, CPEC-LSE, 14 October (Table 2, column “% of pop living in care homes”) |
| Proportion of elderly among nursing home residents: Nursing Home Data Compendium 2015 Edition, Department of Health & Human Services USA (Table 3.11.d), retrieved from https://www.cms.gov/Medicare/Provider-Enrollment-and-Certification/CertificationandComplianc/Downloads/nursinghomedatacompendium_508-2015.pdf |
| Comments: Deaths in long-term care facilities based on data from 42 states. Total is derived from extracted percentage and long-term care deaths. |
|  |
| **United States of America (Study: Kalish)** |
| Seroprevalence information: Seroprevalence study and webpages https://covid.cdc.gov/covid-data-tracker/#demographicsovertime and https://www.kff.org/policy-watch/this-week-in-coronavirus-june-11-to-june-17/ (accessed May 5, 2021; used to calculate the number of cases in community-dwelling elderly, see Comments) |
| Age distribution of COVID-19 deaths: Dataset retrieved from https://data.cdc.gov/NCHS/Provisional-COVID-19-Death-Counts-by-Sex-Age-and-W/vsak-wrfu (accessed February 19, 2021) |
| Total number of deaths for the primary date: Worldometers.info (accessed June 1, 2021) |
| COVID-19 deaths in nursing home residents: Kaiser Family Foundation (KFF), https://www.kff.org/policy-watch/this-week-in-coronavirus-june-18-to-june-25/ (Accessed May 5, 2021) |
| Peak 7-day average of COVID-19 mortality: Worldometers.info |
| Population overall and in elderly: "Annual Estimates of the Resident Population for Selected Age Groups by Sex: April 1, 2010 to July 1, 2019", dataset retrieved from https://www.census.gov/data/tables/time-series/demo/popest/2010s-national-detail.html |
| Population in nursing homes: Comas-Herrera A, Zalakaín J, Lemmon E, Henderson D, Litwin C, Hsu AT, Schmidt AE, Arling G and Fernández J-L (2020) Mortality associated with COVID-19 in care homes: international evidence. Article in LTCcovid.org, International Long-Term Care Policy Network, CPEC-LSE, 14 October (Table 2, column “% of pop living in care homes”) |
| Proportion of elderly among nursing home residents: Nursing Home Data Compendium 2015 Edition, Department of Health & Human Services USA (Table 3.11.d), retrieved from https://www.cms.gov/Medicare/Provider-Enrollment-and-Certification/CertificationandComplianc/Downloads/nursinghomedatacompendium_508-2015.pdf |
| Comments:  Deaths in long-term care facilities based on data from 42 states. Total is derived from extracted percentage and long-term care deaths.  The study specifically targeted people not previously diagnosed with COVID-19, and so the total number of infected was corrected. We calculated the number of cases in community-dwelling elderly up to June 20 by retrieving the cumulative number of cases in elderly (≥65) from the COVID-19 Data Tracker at the US CDC (455,585) and subtracting the cumulative number of cases in nursing homes from KFF (240,138), resulting in 215,447 cases. This number was added to the number of infected community-dwelling elderly. |

**Appendix Table 3a.** Reports not included, main analysis (complementary search for explicitly national-level general population studies without high risk of bias and with at least 500 participants aged ≥70 years).

| **DOI or URL** | **Country/location** | **Reason for exclusion** |
| --- | --- | --- |
| 10.1016/j.ejim.2021.01.029 | Vatican City | Conducted in 2021, identified at screening step |
| 10.1016/j.eclinm.2021.101172 | Zimbabwe | Conducted in 2021, identified at screening step |
| <https://www.nicd.ac.za/wp-content/uploads/2021/03/COVID-19-Special-Public-Health-Surveillance-Bulletin-9-12-March-2021_.pdf> | South Africa | Conducted in 2021, identified at screening step |
| 10.3201/eid2712.211465 | South Africa | Conducted in 2021, identified at screening step |
| 10.1101/2021.06.22.21258711 | Poland | Conducted in 2021, identified at screening step |
| 10.1101/2021.10.19.21265219v1.full-text | Peru | Conducted in 2021, identified at screening step |
| 10.1101/2021.03.23.21254169 | Iraq | Conducted in 2021, identified at screening step |
| 10.1101/2021.07.16.21260611v1.full-text | Bangladesh | Conducted in 2021, identified at screening step |
| 10.1101/2021.08.14.21262042 | Austria | Conducted in 2021, identified at screening step |
| 10.1093/cid/ciab626 | United States of America | Seroprevalence confidence interval reaches zero after Gladen-Rogan correction |
| 10.2139/ssrn.3752659 | Canada | Seroprevalence confidence interval reaches zero after Gladen-Rogan correction |
| 10.1101/2021.08.10.21261777v1 | Denmark | Crude seroprevalence less than 1 - test specificity |
| 10.1038/s41591-020-0949-6 | China | Duplicate, identified at screening step |
| 10.20452/pamw.15796 | Poland | Duplicate, identified at screening step |
| https://www.theadvocate.com/baton_rouge/news/coronavirus/article_b499efa0-8983-11ea-a3f1-af936ecb1d2c.html | United States of America | Duplicate, identified at screening step |
| 10.1101/2020.05.01.20087478v1 | United States of America | Duplicate, identified at screening step |
| https://www.wbtv.com/2020/05/29/antibody-testing-continues-burke-county/&ct=ga&cd=CAAYGTIaOTBkZWE5ODk5NGU5MTg1OTpjb206ZW46VVM&usg=AFQjCNEvSO7bk5TLI9MT5-BeF8ILKdseXA | United States of America | Duplicate, identified at screening step |
| https://www.wcax.com/2020/07/17/early-results-of-uvm-research-tracking-covids-spread-in-vermont/&ct=ga&cd=CAAYCzIaOTBkZWE5ODk5NGU5MTg1OTpjb206ZW46VVM&usg=AFQjCNEblNmlKeakOFmkRkZMbbLnjhptxw | United States of America | Duplicate, identified at screening step |
| 10.3390/pathogens10060710 | United States of America | Duplicate, identified at screening step |
| 10.1177/00333549211055137 | United States of America | Duplicate, identified at screening step |
| https://wiadlek.pl/wp-content/uploads/archive/2021/WLek202105116.pdf | Ukraine | Duplicate, identified at screening step |
| 10.1128/JVI.01828-20 | Switzerland | Duplicate, identified at screening step |
| https://www.ne.ch/autorites/DFS/SCSP/medecin-cantonal/maladies-vaccinations/coronaimmunitas/Pages/R%c3%a9sultats-de-l'%c3%a9tude.aspx | Switzerland | Duplicate, identified at screening step |
| 10.1101/2021.07.31.21261428v2 | Russia | Duplicate, identified at screening step |
| 10.1016/S2214-109X(21)00386-7 | Ethiopia | Duplicate, identified at screening step |
| https://www.thelancet.com/journals/lanmic/article/PIIS2666-5247(20)30053-7/fulltext | China | Duplicate, identified at screening step |
| https://pitangueiras.sp.gov.br/wp-content/uploads/2020/12/Relatorio-Inquerito-COVID-19-Final.pdf | Brazil | Duplicate, identified at screening step |
| 10.1007/s12020-021-02728-8 | Brazil | Duplicate, identified at screening step |
| 10.2196/30406 | Brazil | Duplicate, identified at screening step |
| 10.1111/1753-6405.13155 | Australia | High risk of bias |
| 10.3201/eid2702.204088 | Japan | High risk of bias |
| https://www.haaretz.com/israel-news/.premium-up-to-270-000-israelis-had-coronavirus-new-study-concludes-1.8888435&ct=ga&cd=CAAYETIaOTBkZWE5ODk5NGU5MTg1OTpjb206ZW46VVM&usg=AFQjCNExqw1wlJNVBFrKKUd7hGTplMnR5A | Israel | High risk of bias |
| https://wellcomeopenresearch.org/articles/6-173 | Ghana | High risk of bias |
| 10.3390/pathogens10060774 | Croatia | High risk of bias |
| 10.1101/2020.09.09.20191296v1 | United States of America | High risk of bias |
| 10.1093/cid/ciab519 | United States of America | High risk of bias |
| 10.3390/v13081648 | Russia | COVID-19 mortality data unavailable for elderly at study location |
| 10.1101/2020.08.10.20171942v1 | Brazil | COVID-19 mortality data unavailable for elderly at study location |
| 10.1016/j.ijid.2021.08.028 | Ethiopia | COVID-19 mortality data unavailable for elderly at study location |
| 10.1016/j.cmi.2021.06.002 | Iran | COVID-19 mortality data unavailable for elderly at study location |
| https://www.croiconference.org/abstract/us-population-based-survey-of-vaccine-willingness-and-sars-cov-2-antibody-prevalence/ | United States of America | No seroprevalence estimate for elderly |
| 10.1007/s10654-021-00749-1 | Israel | Following comments arising during peer-review not considered general population sample |
| 10.15388/Amed.2020.28.1.2 | Lithuania | Following comments arising during peer-review not considered general population sample |

**Appendix Table 3b.** Reports not included, sensitivity analysis (original search for studies that sampled or potentially sampled ≥1000 participants aged ≥70 years).

| **DOI or URL** | **Country** | **Reason for exclusion** |
| --- | --- | --- |
| 10.1101/2020.08.10.20171942v1 | Brazil | COVID-19 mortality data unavailable for elderly at study location |
| https://pubmed.ncbi.nlm.nih.gov/33883260/ | China | COVID-19 mortality data unavailable for elderly at study location |
| 10.1016/j.ijid.2021.05.043 | India | COVID-19 mortality data unavailable for elderly at study location |
| 10.3390/diagnostics11030483 | Italy | COVID-19 mortality data unavailable for elderly at study location |
| 10.2144/fsoa-2020-0203 | Italy | COVID-19 mortality data unavailable for elderly at study location |
| 10.19191/EP20.5-6.S2.119 | Italy | COVID-19 mortality data unavailable for elderly at study location |
| 10.31662/jmaj.2020-0094 | Japan | COVID-19 mortality data unavailable for elderly at study location |
| 10.3390/v13081648 | Russia | COVID-19 mortality data unavailable for elderly at study location |
| https://www.rtve.es/noticias/20200617/test-serologicos-torrejon-ardoz-arrojan-2018-habitantes-tiene-anticuerpos-coronavirus/2018787.shtml | Spain | COVID-19 mortality data unavailable for elderly at study location |
| 10.1016/j.xkme.2021.01.002 | USA | COVID-19 mortality data unavailable for elderly at study location |
| 10.1016/j.diagmicrobio.2020.115128 | USA | COVID-19 mortality data unavailable for elderly at study location |
| 10.1111/1753-6405.13155 | Australia | No seroprevalence estimate for elderly |
| 10.1038/s41591-020-0949-6 | China | No seroprevalence estimate for elderly |
| https://www.ifo.de/en/publikationen/2020/monograph-authorship/die-deutschen-und-corona | Germany | No seroprevalence estimate for elderly |
| 10.1093/trstmh/trab109 | India | No seroprevalence estimate for elderly |
| https://www.researchsquare.com/article/rs-80259/v1 | India | No seroprevalence estimate for elderly |
| 10.2188/jea.JE20210324 | Japan | No seroprevalence estimate for elderly (only for a time period in 2021, ineligible) |
| 10.1038/s41598-021-89236-x | Spain | No seroprevalence estimate for elderly |
| https://www.sll.se/verksamhet/halsa-och-vard/nyheter-halsa-och-vard/2020/09/25-september-lagesrapport-om-arbetet-med-det-nya-coronaviruset/ | Sweden | No seroprevalence estimate for elderly; not general population |
| https://www.sll.se/verksamhet/halsa-och-vard/nyheter-halsa-och-vard/2020/09/29-september-lagesrapport-om-covid-19/ | Sweden | No seroprevalence estimate for elderly; not general population |
| https://www.sll.se/verksamhet/halsa-och-vard/nyheter-halsa-och-vard/2020/10/6-oktober-lagesrapport-om-covid-19/ | Sweden | No seroprevalence estimate for elderly; not general population |
| https://www.sll.se/verksamhet/halsa-och-vard/nyheter-halsa-och-vard/2020/10/13-oktober-lagesrapport-om-covid-19/ | Sweden | No seroprevalence estimate for elderly; not general population |
| https://www.sll.se/verksamhet/halsa-och-vard/nyheter-halsa-och-vard/2020/10/20-oktober-lagesrapport-om-covid-19/ | Sweden | No seroprevalence estimate for elderly; not general population |
| https://www.sll.se/verksamhet/halsa-och-vard/nyheter-halsa-och-vard/2020/10/27-oktober-lagesrapport-om-covid-19/ | Sweden | No seroprevalence estimate for elderly; not general population |
| https://www.sll.se/verksamhet/halsa-och-vard/nyheter-halsa-och-vard/2020/11/3-november-lagesrapport-om-covid-19/ | Sweden | No seroprevalence estimate for elderly; not general population |
| https://www.sll.se/verksamhet/halsa-och-vard/nyheter-halsa-och-vard/2020/11/10-november-lagesrapport-om-covid-19/ | Sweden | No seroprevalence estimate for elderly; not general population |
| https://www.sll.se/verksamhet/halsa-och-vard/nyheter-halsa-och-vard/2020/11/17-november-lagesrapport-om-covid-19/ | Sweden | No seroprevalence estimate for elderly; not general population |
| https://www.sll.se/verksamhet/halsa-och-vard/nyheter-halsa-och-vard/2020/11/24-november-lagesrapport-om-covid-19/ | Sweden | No seroprevalence estimate for elderly; not general population |
| https://www.sll.se/verksamhet/halsa-och-vard/nyheter-halsa-och-vard/2020/12/1-december-lagesrapport-om-covid-19/ | Sweden | No seroprevalence estimate for elderly; not general population |
| https://www.sll.se/verksamhet/halsa-och-vard/nyheter-halsa-och-vard/2020/12/8-december-lagesrapport-om-covid-19/ | Sweden | No seroprevalence estimate for elderly; not general population |
| https://www.sll.se/verksamhet/halsa-och-vard/nyheter-halsa-och-vard/2020/12/15-december-lagesrapport-om-covid-19/ | Sweden | No seroprevalence estimate for elderly; not general population |
| https://www.sll.se/verksamhet/halsa-och-vard/nyheter-halsa-och-vard/2020/12/22-december-lagesrapport-om-covid-19/ | Sweden | No seroprevalence estimate for elderly; not general population |
| https://www.sll.se/verksamhet/halsa-och-vard/nyheter-halsa-och-vard/2020/12/29-december-lagesrapport-om-covid-19/ | Sweden | No seroprevalence estimate for elderly; not general population |
| https://www.ons.gov.uk/peoplepopulationandcommunity/healthandsocialcare/conditionsanddiseases/articles/coronaviruscovid19infectionsinthecommunityinengland/december2020 | UK | No seroprevalence estimate for elderly (only for a time period in 2021, ineligible) |
| 10.1016/j.tmrv.2021.07.001 | USA | No seroprevalence estimate for elderly |
| 10.1093/infdis/jiab514 | USA | No seroprevalence estimate for elderly |
| 10.3390/jcm10194341 | USA | No seroprevalence estimate for elderly; not explicitly aiming to generate sample reflecting general population |
| 10.1016/j.annepidem.2020.06.004 | USA | No seroprevalence estimate for elderly |
| 10.1038/s41586-020-2912-6 | USA | No seroprevalence estimate for elderly |
| 10.1001/jama.2020.18598 | USA | No seroprevalence estimate for elderly |
| https://ejmcm.com/article_9453.html | Uzbekistan | No seroprevalence estimate for elderly; not general population |
| 10.1016/j.cmi.2020.09.044 | China | Targets high-risk participants / Does not explicitly aim to generate sample reflecting general population |
| 10.1111/all.14622 | China | Targets high-risk participants / Does not explicitly aim to generate sample reflecting general population |
| 10.1101/2020.08.10.20171850v1 | Denmark | Targets high-risk participants / Does not explicitly aim to generate sample reflecting general population |
| 10.1016/j.revmed.2021.10.330 | France | Targets high-risk participants / Does not explicitly aim to generate sample reflecting general population |
| 10.1101/2021.03.19.21253429 | India | Targets high-risk participants / Does not explicitly aim to generate sample reflecting general population |
| 10.1001/jamanetworkopen.2021.15699 | Italy | Targets high-risk participants / Does not explicitly aim to generate sample reflecting general population |
| 10.1101/2021.01.05.21249247 | Qatar | Targets high-risk participants / Does not explicitly aim to generate sample reflecting general population |
| 10.1093/ageing/afab096 | Spain | Targets high-risk participants / Does not explicitly aim to generate sample reflecting general population |
| 10.1016/S0140-6736(21)00675-9 | UK | Targets high-risk participants / Does not explicitly aim to generate sample reflecting general population |
| 10.1371/journal.pone.0252818 | USA | Targets high-risk participants / Does not explicitly aim to generate sample reflecting general population |
| https://academic.oup.com/jid/advance-article/doi/10.1093/infdis/jiab200/6219118 | USA | Targets high-risk participants / Does not explicitly aim to generate sample reflecting general population |
| 10.1093/cid/ciab519 | USA | Targets high-risk participants / Does not explicitly aim to generate sample reflecting general population |
| 10.1001/jamainternmed.2021.0366 | USA | Targets high-risk participants / Does not explicitly aim to generate sample reflecting general population |
| 10.1016/j.mayocp.2021.03.015 | USA | Targets high-risk participants / Does not explicitly aim to generate sample reflecting general population |
| 10.1093/cid/ciaa1684 | USA | Targets high-risk participants / Does not explicitly aim to generate sample reflecting general population |
| 10.1001/jama.2020.14765 | USA | Targets high-risk participants / Does not explicitly aim to generate sample reflecting general population |
| 10.1016/j.isci.2021.102489 | USA | US study without adjustment for race or ethnicity |
| https://jamanetwork.com/journals/jamanetworkopen/fullarticle/2777502?utm_source=For_The_Media&utm_medium=referral&utm_campaign=ftm_links&utm_term=031621 | USA | US study without adjustment for race or ethnicity |
| 10.1101/2021.01.27.21250615 | USA | US study without adjustment for race or ethnicity |
| 10.1101/2020.09.09.20191296v1 | USA | US study without adjustment for race or ethnicity |
| 10.3390/vaccines9050504 | Greece | Seroprevalence confidence interval reaches zero |
| 10.1101/2021.08.10.21261777v1 | Denmark | Crude seroprevalence less than 1 - test specificity |
| 10.2139/ssrn.3752659 | Canada | Seroprevalence confidence interval reaches zero after Gladen-Rogan correction |
| https://www.sciencedirect.com/science/article/pii/S266677622100096X | Andorra | Duplicate |
| 10.1101/2020.06.08.20125179v5 | Belgium | Duplicate |
| 10.1038/s41598-021-92775-y | Brazil | Duplicate with excluded study (EPICOVID19) |
| https://bit.ly/Epicovid19BRfases1-3 | Brazil | Duplicate/overlapping (10.1016/S2214-109X(20)30387-9) |
| 10.1101/2020.08.10.20171942v1 | Brazil | Duplicate/overlapping (10.1016/S2214-109X(20)30387-9) |
| https://www.covid19immunitytaskforce.ca/wp-content/uploads/2021/01/COVID-19-October-Report-for-CITF.pdf | Canada | Duplicate |
| 10.1111/trf.16296 | Canada | Duplicate |
| 10.1007/s10654-020-00716-2 | France | Duplicate |
| 10.1093/ije/dyab110 | France | Duplicate |
| 10.1016/j.idnow.2020.12.007 | France | Duplicate |
| 10.1101/2021.11.14.21265758 | India | Duplicate |
| 10.1016/S2214-109X(20)30544-1 | India | Duplicate |
| https://www.istat.it/it/files//2020/08/ReportPrimiRisultatiIndagineSiero.pdf | Italy | Duplicate |
| 10.1016/j.isci.2021.102646 | Qatar | Duplicate |
| 10.1101/2021.03.11.21253142v1 | Spain | Duplicate |
| 10.1016/j.lanepe.2021.100098 | UK | Duplicate |
| https://www.ukbiobank.ac.uk/media/x0nd5sul/ukb_serologystudy_report_revised_6months_jan21.pdf | UK | Duplicate |
| 10.1101/2020.10.26.20219725v1.full.pdf | UK | Duplicate |
| 10.1126/scitranslmed.abh3826 | USA | Duplicate |
| 10.1101/2020.11.10.20215145 | USA | Duplicate |

Note. The duplicate records shown in this table are not a comprehensive list of duplicates found.

**Appendix Table 4**. Seroprevalence estimate corrections for test performance using the Gladen-Rogan formula.

| **First author last name** | **Assay sensitivity** | **Assay specificity** | **Seroprevalence description** | **Seroprevalence (%)** | **CI lower bound (%)** | **CI upper bound (%)** | **Gladen-Rogan corrected seroprevalence (%)** | **Gladen-Rogan corrected CI lower bound (%)** | **Gladen-Rogan corrected CI upper bound (%)** |
| --- | --- | --- | --- | --- | --- | --- | --- | --- | --- |
| Abu-Raddad | 97.92 | 99.95 | 70-79 | 13.9 |  |  | 14.2 |  |  |
| Abu-Raddad | 97.92 | 99.95 | 80+ | 9.8 |  |  | 10.01 |  |  |
| Abu-Raddad | 97.92 | 99.95 | Adjusted, entire elderly group |  | 11.5 | 14.7 |  | 11.75 | 15.02 |
| Abu-Raddad | 97.92 | 99.95 | Crude, entire elderly group | 8.96 | 7.68 | 10.37 | 9.15 | 7.85 | 10.6 |
| Abu-Raddad | 97.92 | 99.95 | 0-9 | 5.5 |  |  | 5.62 |  |  |
| Abu-Raddad | 97.92 | 99.95 | 10-19 | 6.7 |  |  | 6.85 |  |  |
| Abu-Raddad | 97.92 | 99.95 | 20-29 | 23.1 |  |  | 23.6 |  |  |
| Abu-Raddad | 97.92 | 99.95 | 30-39 | 25.3 |  |  | 25.85 |  |  |
| Abu-Raddad | 97.92 | 99.95 | 40-49 | 30.1 |  |  | 30.75 |  |  |
| Abu-Raddad | 97.92 | 99.95 | 50-59 | 30.4 |  |  | 31.06 |  |  |
| Abu-Raddad | 97.92 | 99.95 | 60-69 | 23.9 |  |  | 24.42 |  |  |
| Anand | 100 | 99.8 | 65-79 | 8.3 | 7.8 | 8.9 | 8.31 | 7.82 | 8.92 |
| Anand | 100 | 99.8 | 80+ | 7.4 | 6.5 | 8.5 | 7.41 | 6.51 | 8.52 |
| Anand | 100 | 99.8 | Crude, entire elderly group | 7.64 |  |  | 7.65 |  |  |
| Anand | 100 | 99.8 | 18-44 | 9.8 |  |  | 9.82 |  |  |
| Anand | 100 | 99.8 | 45-64 | 9.5 |  |  | 9.52 |  |  |
| Charlton | 66.7 | 100 | 70-79 | 1.19 | 0.69 | 1.7 | 1.79 | 1.03 | 2.55 |
| Charlton | 66.7 | 100 | 80+ | 0.76 | 0.23 | 1.28 | 1.13 | 0.35 | 1.91 |
| Charlton | 66.7 | 100 | Crude, entire elderly group | 1.03 |  |  | 1.54 |  |  |
| Charlton | 66.7 | 100 | 0-9 | 2.52 |  |  | 3.78 |  |  |
| Charlton | 66.7 | 100 | 10-19 | 1.87 |  |  | 2.81 |  |  |
| Charlton | 66.7 | 100 | 20-29 | 3.38 |  |  | 5.07 |  |  |
| Charlton | 66.7 | 100 | 30-39 | 3.32 |  |  | 4.98 |  |  |
| Charlton | 66.7 | 100 | 40-49 | 2.16 |  |  | 3.25 |  |  |
| Charlton | 66.7 | 100 | 50-59 | 2.14 |  |  | 3.21 |  |  |
| Charlton | 66.7 | 100 | 60-69 | 1.47 |  |  | 2.2 |  |  |
| ISCII | 89.7 | 100 | 70-74 | 7.6 | 6.4 | 9.1 | 8.47 | 7.13 | 10.14 |
| ISCII | 89.7 | 100 | 75-79 | 7 | 5.6 | 8.7 | 7.8 | 6.24 | 9.7 |
| ISCII | 89.7 | 100 | 80-84 | 6.9 | 5.2 | 9.2 | 7.69 | 5.8 | 10.26 |
| ISCII | 89.7 | 100 | 85-89 | 6.6 | 4.5 | 9.5 | 7.36 | 5.02 | 10.59 |
| ISCII | 89.7 | 100 | 90+ | 6.5 | 3.9 | 10.8 | 7.25 | 4.35 | 12.04 |
| ISCII | 89.7 | 100 | 0-4 | 4.3 |  |  | 4.79 |  |  |
| ISCII | 89.7 | 100 | 5-9 | 5.9 |  |  | 6.58 |  |  |
| ISCII | 89.7 | 100 | 10-14 | 6.8 |  |  | 7.58 |  |  |
| ISCII | 89.7 | 100 | 15-19 | 6.1 |  |  | 6.8 |  |  |
| ISCII | 89.7 | 100 | 20-24 | 8.1 |  |  | 9.03 |  |  |
| ISCII | 89.7 | 100 | 25-29 | 7.6 |  |  | 8.47 |  |  |
| ISCII | 89.7 | 100 | 30-34 | 7 |  |  | 7.8 |  |  |
| ISCII | 89.7 | 100 | 35-39 | 6.7 |  |  | 7.47 |  |  |
| ISCII | 89.7 | 100 | 40-44 | 6.6 |  |  | 7.36 |  |  |
| ISCII | 89.7 | 100 | 45-49 | 7.3 |  |  | 8.14 |  |  |
| ISCII | 89.7 | 100 | 50-54 | 8.5 |  |  | 9.48 |  |  |
| ISCII | 89.7 | 100 | 55-59 | 7.3 |  |  | 8.14 |  |  |
| ISCII | 89.7 | 100 | 60-64 | 7.8 |  |  | 8.7 |  |  |
| ISCII | 89.7 | 100 | 65-69 | 7 |  |  | 7.8 |  |  |
| Merkely | 66.7 | 100 | Adjusted, entire elderly group | 0.75 | 0.21 | 1.29 | 1.12 | 0.31 | 1.93 |
| Merkely | 66.7 | 100 | Crude, entire elderly group | 0.62 |  |  | 0.93 |  |  |
| Merkely | 66.7 | 100 | 14-39 | 0.56 |  |  | 0.84 |  |  |
| Merkely | 66.7 | 100 | 40-64 | 0.7 |  |  | 1.05 |  |  |
| Paulino-Ramirez | 56.7 | 100 | Crude, entire elderly group | 5.97 | 5.13 | 6.94 | 10.53 | 9.05 | 12.24 |
| Paulino-Ramirez | 56.7 | 100 | 0-9 | 8.64 |  |  | 15.24 |  |  |
| Paulino-Ramirez | 56.7 | 100 | 10-19 | 3.85 |  |  | 6.79 |  |  |
| Paulino-Ramirez | 56.7 | 100 | 20-29 | 4.6 |  |  | 8.11 |  |  |
| Paulino-Ramirez | 56.7 | 100 | 30-39 | 5.09 |  |  | 8.98 |  |  |
| Paulino-Ramirez | 56.7 | 100 | 40-49 | 5.92 |  |  | 10.44 |  |  |
| Paulino-Ramirez | 56.7 | 100 | 50-59 | 6.09 |  |  | 10.74 |  |  |
| Reicher | 76.7 | 99.9 | 70+ | 1.7 | 1.4 | 2 | 2.22 | 1.83 | 2.61 |
| Reicher | 76.7 | 99.9 | 0-9 | 5.6 |  |  | 7.3 |  |  |
| Reicher | 76.7 | 99.9 | 10-19 | 7.8 |  |  | 10.17 |  |  |
| Reicher | 76.7 | 99.9 | 20-29 | 4.8 |  |  | 6.26 |  |  |
| Reicher | 76.7 | 99.9 | 30-39 | 3.7 |  |  | 4.82 |  |  |
| Reicher | 76.7 | 99.9 | 40-49 | 4.1 |  |  | 5.35 |  |  |
| Reicher | 76.7 | 99.9 | 50-59 | 3.7 |  |  | 4.82 |  |  |
| Reicher | 76.7 | 99.9 | 60-69 | 2.6 |  |  | 3.39 |  |  |
| Royo-Cebrecos | 90.6 | 99.2 | Crude, entire elderly group | 13.41 | 12.41 | 14.46 | 14.92 | 13.82 | 16.1 |
| Warszawski, INSERM | 80 | 99.6 | 65-74 | 4.3 | 3.8 | 4.9 | 5.4 | 4.77 | 6.16 |
| Warszawski, INSERM | 80 | 99.6 | 75+ | 3.7 | 2.9 | 4.7 | 4.64 | 3.64 | 5.9 |
| Warszawski, INSERM | 80 | 99.6 | Crude, entire elderly group | 4.2 |  |  | 5.27 |  |  |
| Warszawski, INSERM | 80 | 99.6 | 15-17 | 9.8 |  |  | 12.31 |  |  |
| Warszawski, INSERM | 80 | 99.6 | 25-34 | 7.2 |  |  | 9.04 |  |  |
| Warszawski, INSERM | 80 | 99.6 | 35-44 | 6.5 |  |  | 8.16 |  |  |
| Warszawski, INSERM | 80 | 99.6 | 45-54 | 6.5 |  |  | 8.16 |  |  |
| Warszawski, INSERM | 80 | 99.6 | 55-64 | 5.3 |  |  | 6.65 |  |  |

Assay performance estimates are based on https://www.ncbi.nlm.nih.gov/pmc/articles/PMC7336910/ for Merkely, based on https://pubmed.ncbi.nlm.nih.gov/34461153/ for Abu-Raddad, based on https://www.coronavirus-diagnostics.com/documents/Indications/Infections/Coronavirus/YI_2606_I_UK_C.pdf for Warszawski (sensitivity is given as 43.7% in the first 10 days and 94.4% after 10 days and the survey measurements were in the middle of a peak of the second wave, therefore 80% sensitivity has been imputed), based on https://www.ncbi.nlm.nih.gov/pmc/articles/PMC7336910/ for Charlton (of note, all positives were verified with a second method, so specificity=100% indeed), based on information provided by the serosurvey authors in their article for Anand, for ISCII, Reicher, and for Paulino-Ramirez, and based on vendor data for Royo-Cebrecos.

Of note, assay performance can vary in different evaluations and settings and it also carries substantial uncertainty. This may also affect the eligibility of the Hungary study (Merkely et al.) that has very low seroprevalence, if specificity is 99.3% instead of 100%. Moreover, typically assay sensitivity is evaluated using as positive gold standard samples from patients with positive PCR. In the early months of the pandemic (when these assay evaluations were performed) typically these were patients with overt symptoms. Sensitivity in asymptomatic individuals who had not been detected by PCR may be lower. If so, the corrected seroprevalence may be underestimated and IFR overestimated. Use of multiple types of antibodies (IgG/IgM/IgA) and correction of seroprevalence when not all antibody types have been assessed may counter some of this effect and this is why we had adopted this correction process in our original protocol, but, as requested by a reviewer, eventually it was not adopted in the main analysis. Assay specificity is typically evaluated using as negative gold standard samples from patients without a COVID-19 diagnosis. However, many infections were missed; if missed infected people have contaminated the negative control sample, specificity would be underestimated. If so, the corrected seroprevalence may be underestimated and IFR overestimated.

**Appendix Table 5.** Uncorrected and corrected (for unmeasured antibody types or for seroreversion) infection fatality rate in community-dwelling elderly

| **Location (First author)** | **Antibody types measured** | **Study midpoint (2020)*** | **Peak of wave 1^†^** | **Time lag between primary date and peak (months)** | **IFR in community-dwelling elderly (uncorrected)** | **IFR in community-dwelling elderly (corrected for unmeasured antibody types)** | **IFR in community-dwelling elderly, corrected for 1% relative seroreversion/month^‡^** | **IFR in community-dwelling elderly, corrected for 5% relative seroreversion/month^‡^** | **IFR in community-dwelling elderly, corrected for 10% relative seroreversion/month^‡^** |
| --- | --- | --- | --- | --- | --- | --- | --- | --- | --- |
| ***Main analysis*** | | | | | | | | | |
| Andorra (Royo-Cebrecos)** | IgG/IgM | 2020-05-16 | 2020-04-01 | 1.48 | 2.02 | 1.82 | 1.99 | 1.87 | 1.73 |
| Ontario, Canada (Public Health Ontario, COVID-19 Immunity Task Force) | IgG | 2020-06-17 | 2020-04-26 | 1.71 | 1.96 | 1.59 | 1.93 | 1.8 | 1.64 |
| Denmark (Espenhain) | IgG/IgM/IgA | 2020-09-19 | 2020-03-31 | 5.65 | 2.92 | 2.92 | 2.76 | 2.19 | 1.61 |
| France (Warszawski, INSERM)** | IgG | 2020-11-24 | 2020-11-11 | 0.43 | 4.14 | 3.35 | 4.12 | 4.05 | 3.96 |
| Ile-de-France, France (Carrat) | IgG | 2020-05-14 | 2020-03-29 | 1.51 | 7.1 | 5.75 | 7 | 6.57 | 6.06 |
| Nouvelle-Aquitaine, France (Carrat) | IgG | 2020-05-14 | 2020-03-29 | 1.51 | 1.27 | 1.03 | 1.25 | 1.18 | 1.08 |
| Hungary (Merkely)** | IgG | 2020-05-08 | 2020-04-16 | 0.72 | 1.85 | 1.5 | 1.83 | 1.78 | 1.71 |
| Iceland (Gudbjartsson) | IgG/IgM/IgA | 2020-05-25 | 2020-04-02 | 1.74 | 3.12 | 3.12 | 3.06 | 2.85 | 2.59 |
| Italy (ISTAT) | IgG | 2020-06-19 | 2020-03-26 | 2.79 | 7.63 | 6.18 | 7.42 | 6.61 | 5.69 |
| Netherlands (Vos) | IgG | 2020-06-14 | 2020-04-01 | 2.43 | 2.27 | 1.84 | 2.22 | 2 | 1.76 |
| Spain (ISCII)** | IgG | 2020-11-22 | 2020-03-27 | 7.89 | 4.55 | 3.68 | 4.2 | 3.03 | 1.98 |
| England (Ward) | IgG | 2020-07-02 | 2020-04-03 | 2.96 | 9.68 | 7.84 | 9.4 | 8.32 | 7.09 |
| USA (Kalish) | IgG/IgM/IgA | 2020-06-20 | 2020-04-14 | 2.2 | 2.27 | 2.27 | 2.22 | 2.03 | 1.8 |
| ***Sensitivity analysis (Non-peer-reviewed pre-specified selection criteria)*** | | | | | | | | | |
| Belgium (Herzog) | IgG | 2020-04-02 | 2020-04-07 | -0.16 | 3.79 | 3.07 | 3.79 | 3.79 | 3.79 |
| Canada (Saeed, Canadian Blood Services) | IgG | 2020-06-13 | 2020-04-30 | 1.45 | 3.23 | 2.62 | 3.18 | 3 | 2.77 |
| Alberta, Canada (Charlton)** | IgG | 2020-12-08 | 2020-04-13 (2020-12-27) **^††^** | 7.85 (-0.4) **^††^** | 4.8 | 3.89 | 4.44 (4.8) **^††^** | 3.21 (4.8) **^††^** | 2.1 (4.8) **^††^** |
| Denmark (Pedersen) | IgG/IgM/IgA | 2020-06-10 | 2020-03-31 | 2.33 | 4.43 | 4.43 | 4.33 | 3.93 | 3.47 |
| Dominican Republic (Paulino-Ramirez)** | IgG | 2020-05-15 | 2020-04-05 | 1.31 | 0.19 | 0.16 | 0.19 | 0.18 | 0.17 |
| India (Murhekar) | IgG | 2020-09-04 | 2020-09-10 | -0.2 | 0.43 | 0.35 | 0.43 | 0.43 | 0.43 |
| Tamil Nadu, India (Malani) | IgG | 2020-11-09 | 2020-07-18 | 3.75 | 0.32 | 0.26 | 0.31 | 0.27 | 0.22 |
| Israel (Reicher)** | IgG | 2020-07-09 | 2020-04-12 | 2.89 | 1.12 | 0.91 | 1.09 | 0.97 | 0.83 |
| Qatar (Abu-Raddad)** | IgG | 2020-06-28 | 2020-06-23 | 0.16 | 2.18 | 1.76 | 2.17 | 2.16 | 2.14 |
| UK (UK Biobank) | Missing/Unclear | 2020-07-05 | 2020-04-06 | 2.96 | 3.53 | 3.53 | 3.43 | 3.04 | 2.59 |
| England and Wales (Public Health England) | Missing/Unclear | 2020-05-15 | 2020-04-03 | 1.38 | 7.55 | 7.55 | 7.45 | 7.04 | 6.53 |
| Greater Glasgow and Clyde, Scotland (Hughes) | IgG | 2020-04-19 | 2020-04-13 | 0.2 | 2.87 | 2.32 | 2.86 | 2.84 | 2.81 |
| USA (Anand)** | IgG/IgM/IgA | 2020-07-07 | 2020-04-14 | 2.76 | 1.2 | 1.2 | 1.17 | 1.05 | 0.9 |

Results of seroreversion analyses are exploratory and the exact rate of seroreversion may differ according to assay and threshold used for a positive reading.

* Midpoint of the seroprevalence sampling period. † The peak of the first epidemic wave was defined as one week before the date with the highest rolling average 7-day mortality (according to Worldometer, situational reports, or Wikipedia). The first epidemic wave was defined to end by the date with the lowest 7-day average of daily deaths since the beginning of the epidemic. If two or more dates were tied for peak values, we chose the date corresponding to the midpoint between the first and last one. ‡ Seroreversion correction of the IFR by X^m^-fold, where m is the number of months from the peak of the first epidemic wave in the specific location and X is given values of 0.99, 0.95, and 0.90 corresponding to 1%, 5%, and 10% relative rate of seroreversion every month, respectively.** Seroprevalence corrected for test performance with the Gladen-Rogan formula. **^††^** For Alberta, Canada, main estimates are based on the peak of the first wave; estimates based on peak of the second wave are given within parentheses.

**Appendix Table 6**. Sensitivity analysis with a later cutoff for cumulative COVID-19 mortality (study midpoint plus two weeks instead of plus one week).

| **Location (First author)** | **Closest date to study midpoint + 1 week, with total deaths available** | **Closest date to study midpoint + 2 weeks, with total deaths available** | ***Relative* increase in IFR from cutoff +1 week to cutoff +2 weeks after study midpoint (%)** | **Corrected IFR in community-dwelling elderly, using study midpoint + 1 week (%)** | **Corrected IFR in community-dwelling elderly, using study midpoint + 2 weeks (%)** |
| --- | --- | --- | --- | --- | --- |
| ***Main analysis*** |  |  |  |  |  |
| Andorra (Royo-Cebrecos)* | 2020-05-23 | 2020-05-30 | 0 | 2.02 | 2.02 |
| Ontario, Canada (Public Health Ontario, COVID-19 Immunity Task Force) | 2020-06-24 | 2020-07-01 | 1.71 | 1.96 | 1.99 |
| Denmark (Espenhain) | 2020-09-25 | 2020-10-02 | 1.4 | 2.92 | 2.96 |
| France (Warszawski, INSERM)* | 2020-12-02 | 2020-12-09 | 5.32 | 4.14 | 4.36 |
| Ile-de-France, France (Carrat) | 2020-05-26 | 2020-05-28 | Unavailable | 7.1 | 7.1 |
| Nouvelle-Aquitaine, France (Carrat) | 2020-05-25 | 2020-05-28 | Unavailable | 1.27 | 1.27 |
| Hungary (Merkely)* | 2020-05-15 | 2020-05-22 | 7.69 | 1.85 | 1.99 |
| Iceland (Gudbjartsson) | 2020-06-01 | 2020-06-08 | 0 | 3.12 | 3.12 |
| Italy (ISTAT) | 2020-06-26 | 2020-07-03 | 0.34 | 7.63 | 7.66 |
| Netherlands (Vos) | 2020-06-21 | 2020-06-28 | 0.25 | 2.27 | 2.28 |
| Spain (ISCII)* | 2020-11-29 | 2020-12-06 | 3.13 | 4.55 | 4.69 |
| England (Ward) | 2020-07-10 | 2020-07-17 | 5.57 | 9.68 | 10.22 |
| USA (Kalish) | 2020-06-27 | 2020-07-04 | 2.84 | 2.27 | 2.34 |
| ***Sensitivity analysis (Non-peer-reviewed pre-specified selection criteria)*** |  |  |  |  |  |
| Belgium (Herzog) | 2020-04-09 | 2020-04-16 | 56.51 | 3.79 | 5.93 |
| Canada (Saeed, Canadian Blood Services) | 2020-06-14 | 2020-06-29 | 5.16 | 3.23 | 3.4 |
| Alberta, Canada (Charlton)* | 2020-12-15 | 2020-12-22 | 5.85 | 4.8 | 5.08 |
| Denmark (Pedersen) | 2020-06-22 | 2020-06-24 | 0.84 | 4.43 | 4.47 |
| Dominican Republic (Paulino-Ramirez)* | 2020-05-22 | 2020-05-29 | 7.02 | 0.19 | 0.21 |
| India (Murhekar) | 2020-11-20 | 2020-09-18 | 10.46 | 0.43 | 0.48 |
| Tamil Nadu, India (Malani) | 2020-11-20 | 2020-11-23 | Unavailable | 0.32 | 0.32 |
| Israel (Reicher)* | 2020-07-16 | 2020-07-23 | 15.1 | 1.12 | 1.29 |
| Qatar (Abu-Raddad)* | 2020-07-05 | 2020-07-12 | 14.84 | 2.18 | 2.5 |
| UK (UK Biobank) | 2020-07-10 | 2020-07-19 | 0.37 | 3.53 | 3.55 |
| England and Wales (Public Health England) | 2020-05-22 | 2020-05-29 | 3.92 | 7.55 | 7.85 |
| Greater Glasgow and Clyde, Scotland (Hughes) | 2020-04-26 | 2020-05-03 | 23.03 | 2.87 | 3.53 |
| USA (Anand)* | 2020-07-14 | 2020-07-21 | 4.27 | 1.2 | 1.26 |

* Seroprevalence corrected for test performance with the Gladen-Rogan formula.

**Appendix Text 1:** Comparison against other studies estimating IFR

We present here comparisons against studies that have estimated age-stratified IFR: Levin et al. (1), the US CDC pandemic planning scenarios (2) (practically based on Levin et al.), Driscoll et al (3), and the Imperial College COVID-19 response team (4).

To allow for a full comparison across age groups, we have also extracted data for younger age strata. This allows to tell whether our lower estimates of IFR in the elderly extend to a similar discrepancy also in other age groups, or the difference is specific to the elderly. Among the included studies, whenever there were seroprevalence estimates and COVID-19 mortality data available for younger age groups, we complemented data extraction for all available age strata. Studies were excluded if no mortality data were available for any age stratum of maximum width 20 years and maximum age 70 years. We used the same time points as those selected for the elderly data. We included all age strata with a maximum width of 20 years and available COVID-19 mortality information. We corresponded the respective seroprevalence estimates for each age stratum with eligible mortality data. Consecutive strata of 1-5 years were merged to generate 10-year bins. For seroprevalence estimates we used the age strata that most fully covered the age bin for which mortality data were available; for the youngest age groups seroprevalence data from the closest available group with any sampled persons ≤20 years were accepted. E.g. for the Ward et al UK study, eligible age strata were 0-19 (paired with seroprevalence data for 20-24), 20-24, 25-34, 35-44, 45-54, 55-64. Population statistics for each analyzed age bin were obtained from the same sources as for the elderly. For age strata with multiple estimates from the same country, we calculated the sample size-weighted IFR per country before estimating median IFRs across locations for age groups 0-19, 20-29, 30-39, 40-49, 50-59, and 60-69 years. IFR estimates were placed in these age groups according to their midpoint, regardless of whether they perfectly matched the age group or not, e.g. an IFR estimate for age 18-29 years was placed in the 20-29 years group. As for the main analysis, following a suggestion from peer-reviewers, whenever no adjustment was made for test performance, we adjusted the estimates for test performance using the Gladen-Rogan formula. No correction was made for types of unmeasured antibodies.

The figure below shows the IFRs in younger age groups derived from seroprevalence studies. Sample size weighted IFRs were calculated for countries with multiple estimates available. The asterisk sign (*) denotes values produced by zero death counts in that age bracket. The median IFR was 0.0009%, 0.012%, 0.035%, 0.109%, 0.34%, and 1.07%, at 0-19, 20-29, 30-39, 40-49, 50-59, and 60-69 years without accounting for seroreversion considering available data from the studies of the main analysis, and 0.001%, 0.010%, 0.023%, 0.050%, 0.15%, and 0.49%, at 0-19, 20-29, 30-39, 40-49, 50-59, and 60-69 years without accounting for seroreversion considering available data from all studies.


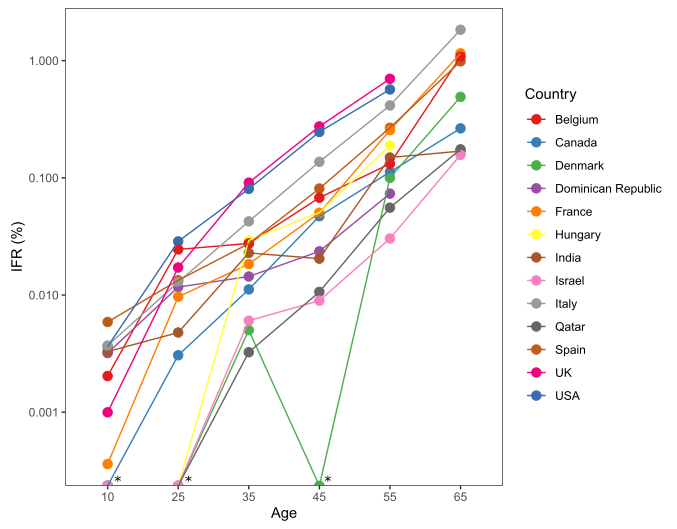


The following figure plots the age-specific infection fatality rates (IFRs) in this work and some previously published overviews (log-scale and normal scale). The eldest groups, which do not have an upper age bound, are placed at the midpoint of their lower bound and 110. Estimates for O’Driscoll regarding age brackets 65 years and older refer to the community-dwelling, while other estimates outside this work refer to all elderly.


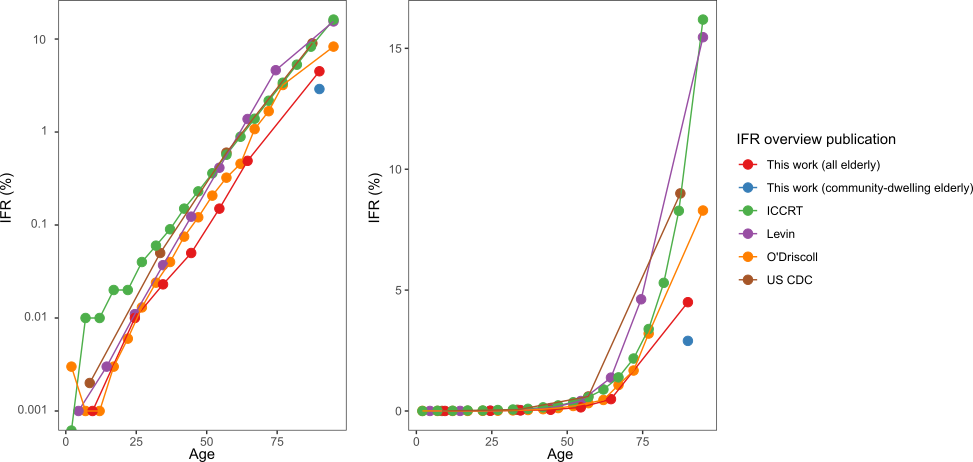


Levin et al (1) is the basis for the US CDC pandemic planning scenarios (2). Levin et al report IFR 4.6% at age 75, and 15% at age 85 without separating nursing home deaths (thus referring to all elderly). Given the age pyramid of the USA population, the CDC operationalization of the Levin et al. estimates infers IFR=9% for all elderly ≥65 years old in their “best current scenario” (2) while our analysis suggests an IFR lower than even the most optimistic CDC planning scenario (IFR=2.6%) and our estimate would be even further decreased if there is substantial seroreversion. For Iceland, operationalization of the Levin et al. estimates would similarly yield very large deviation from our estimates, while our IFR regression estimates for Iceland agree with the empirical IFR in that country which has been calculated based on extremely meticulous characterization of the spread of the infection through aggressive PCR testing performed even in the first wave in addition to serology. Levin et al also estimate about double the IFR compared with our estimates for the age groups 40-49, 50-59, and 60-69. The assessment of Levin et al. was based on relatively sparse data for these age groups and was limited to advanced economies with the highest proportions of very elderly populations among the elderly group. The authors counted deaths four weeks after the midpoint of the seroprevalence sampling period, which is the longest among existing overviews, with the argument that there is large potential reporting lag (although available mortality statistics are commonly updated retrospectively for the date of death). Also, almost all included studies came from hard-hit locations, where IFR may be substantially higher (5). Selection of studies with lower seroprevalence and/or higher death counts may explain why their estimates for middle-aged and elderly are substantially higher than ours.

O’Driscoll et al (3) modeled 22 seroprevalence studies, and carefully comment how outbreaks in nursing homes can drive overall population IFRs. For young and middle-aged groups, their estimates largely agree with those presented here. Their estimates for elderly, which are reflecting the community-dwelling, are still higher than ours. For ages ≥65 years, their model uses data derived from one location (England) on deaths that did not occur in nursing homes and is validated against other locations with such statistics. This may overestimate the community-dwelling proportion of deaths, since deaths of nursing home residents occurring in hospitals are counted in the England community estimates. Conversely our evaluation adds granularity by using deaths in nursing home residents from many countries, and by using seroprevalence estimates from 25 serosurveys with many elderly individuals.

The Imperial College COVID-19 response team (4) presents much higher IFR estimates for elderly overall. They use a very narrowly selected subset of 10 studies in 9 countries, five of which had sampled >1000 elderly people. Their selection criteria required >100 deaths in the location at the seroprevalence study midpoint, which skews the sample towards heavily-hit areas and higher IFRs.

Some published studies also present IFRs in elderly people for single locations based on seroprevalence data, but these are unavoidably location-limited. Most of those studies reporting IFR in the elderly represent locations with the highest reported IFRs for the overall population, thus the reported IFR estimates for the elderly may also be substantially inflated versus the global experience. For example, Pastor-Barriuso *et al* (6) used nationwide estimates from Spain (7) (ENE-COVID study, comprised by our sample (8)) to calculate the IFR in community-dwelling persons, reporting an IFR in individuals 70-79 years old of 4.96% and in those 80 years or older of 11.6% using confirmed COVID-19 deaths. The estimates did not exclude deaths of nursing home residents that occurred in hospitals. Molenberghs *et al* (9) used a statistical model based on seroprevalence (10) and granular national data from Belgium to estimate IFRs for the community-dwelling at 1.2% for individuals 70-79 years, 1.0% for 80-89 years, and 2.4% for 90 years or older (slightly lower than our estimates). Blackburn *et al* (11) calculated the IFR in Indiana, USA, at 1.71% for community-dwelling persons aged 60 years or older. The seroprevalence study used as basis for these calculations (12) was later updated by the same team using statistical methods to address “nonresponse among various demographic groups and to adjust for testing errors to reduce bias in the estimates of the overall disease prevalence”, resulting in an upweighting of individuals identifying as Black or “Other (including multiracial)” non-white, or with Hispanic ethnicity, and an overall seroprevalence of 3.6% instead of 2.8% (13). Overall, the Indiana results are quite consistent with our estimates. Conversely, Mahajan *et al* (14) report the IFR in Connecticut, USA, at 16.46% for community-dwelling persons 65 years or older. This estimate depends on seroprevalence data for very few people >65 (n=187­).

Some studies were excluded because of criteria proposed during peer-review that were not in the original protocol (see detailed tables above with exclusions). At a quick glance their IFR values, when they can be calculated, are similar to the IFR values calculated in the eligible studies included in our analysis. One of the reviewers mentioned specifically a study by Sullivan et al (15) and requested the reasons for exclusion. This study had 1173 enrolled people ≥65 years old, thus expectedly lower than 1000 people ≥70 years old based on the underlying population distribution. It would have been eligible for the sensitivity analysis including studies with 500-1000 eligible elderly, but even for this analysis it was excluded because it found a seroprevalence of 2.48% (95% CI 1.22-4.98%) in the elderly stratum which became 1.86% (0.00-3.81%) after adjustment for test performance, thus the 95% CI does not exclude 0. As a side note, the study was performed in late 2020 on dry blood spots and it found overall seroprevalence of 4.71%, but it acknowledged extensive waning of antibodies over time and in an attempt to adjust for this, it estimated a seroprevalence almost three times higher after conservatively adjusting for waning (11.9%). This illustrates the difficulty of making adjustments for waning antibodies, but it is likely that major adjustment is needed for seroprevalence studies done after the initial few months of the pandemic.

For persons 0-19 years, our median IFR estimates correspond to one death per 111,100 persons with COVID-19 infection, followed by estimates of 1:10,000 in ages 20-29, 1:4000 in ages 30-39, and 1:2000 in ages 40-49. The Imperial College study (5) has ~10 times higher estimates for persons 0-19 years and ~3 times higher for persons 20-29 years old; otherwise estimates in age groups <50 years are fairly consistent across previous (1,4) and current analyses despite methodological differences.

1. Levin AT, Hanage WP, Owusu-Boaitey N, Cochran KB, Walsh SP, Meyerowitz-Katz G. Assessing the age specificity of infection fatality rates for COVID-19: systematic review, meta-analysis, and public policy implications. European journal of epidemiology. 2020;35(12):1123-38.

2. United States Centers for Disease Control and Prevention. COVID-19 Pandemic Planning Scenarios 2021 [updated March 19, 2021. Available from: https://www.cdc.gov/coronavirus/2019-ncov/hcp/planning-scenarios.html.

3. O'Driscoll M, Dos Santos GR, Wang L, Cummings DAT, Azman AS, Paireau J, et al. Age-specific mortality and immunity patterns of SARS-CoV-2. Nature. 2020.

4. Brazeau N, Verity R, Jenks S, et al. COVID-19 Infection Fatality Ratio: Estimates from Seroprevalence. Imperial College London. 2020-10-29.

5. Ioannidis JPA. Infection fatality rate of COVID-19 inferred from seroprevalence data. Bull World Health Organ. 2021;99:19–33F.

6. Pastor-Barriuso R, Pérez-Gómez B, Hernán MA, Pérez-Olmeda M, Yotti R, Oteo-Iglesias J, et al. Infection fatality risk for SARS-CoV-2 in community dwelling population of Spain: nationwide seroepidemiological study. BMJ. 2020;371:m4509.

7. Pollán M, Pérez-Gómez B, Pastor-Barriuso R, Oteo J, Hernán MA, Pérez-Olmeda M, et al. Prevalence of SARS-CoV-2 in Spain (ENE-COVID): a nationwide, population-based seroepidemiological study. Lancet (London, England). 2020;396(10250):535-44.

8. Ministerio de Sanidad, III IdSC. ESTUDIO ENE-COVID: CUARTA RONDA. ESTUDIO NACIONAL DE SERO-EPIDEMIOLOGÍA DE LA INFECCIÓN POR SARS-COV-2 EN ESPAÑA. 15 DE DICIEMBRE DE 2020. <https://wwwmscbsgobes/gabinetePrensa/notaPrensa/pdf/1512151220163348113pdf>; <https://portalcneisciiies/enecovid19/informes/informe_cuarta_rondapdf>. 2021.

9. Molenberghs G, Faes C, Verbeeck J, Deboosere P, Abrams S, Willem L, et al. Belgian COVID-19 Mortality, Excess Deaths, Number of Deaths per Million, and Infection Fatality Rates (9 March — 28 June 2020). medRxiv. 2020:2020.06.20.20136234.

10. Herzog S, De Bie J, Abrams S, Wouters I, Ekinci E, Patteet L, et al. Seroprevalence of IgG antibodies against SARS coronavirus 2 in Belgium – a serial prospective cross-sectional nationwide study of residual samples. medRxiv. 2021:2020.06.08.20125179.

11. Blackburn J, Yiannoutsos CT, Carroll AE, Halverson PK, Menachemi N. Infection Fatality Ratios for COVID-19 Among Noninstitutionalized Persons 12 and Older: Results of a Random-Sample Prevalence Study. Annals of internal medicine. 2020;174(1):135-6.

12. Menachemi N, Yiannoutsos C, Dixon BT, et al. Population Point Prevalence of SARS-CoV-2 Infection Based on a Statewide Random Sample — Indiana, April 25–29, 2020. MMWR Morbidity and mortality weekly report. 2020;69:960-964.

13. Yiannoutsos CT, Halverson PK, Menachemi N. Bayesian estimation of SARS-CoV-2 prevalence in Indiana by random testing. Proceedings of the National Academy of Sciences. 2021;118(5):e2013906118.

14. Mahajan S, Caraballo C, Li SX, Dong Y, Chen L, Huston SK, et al. SARS-CoV-2 Infection Hospitalization Rate and Infection Fatality Rate Among the Non-Congregate Population in Connecticut. The American journal of medicine. 2021.

15. Sullivan PS, Siegler AJ, Shioda K, et al. Severe Acute Respiratory Syndrome Coronavirus 2 Cumulative Incidence, United States, August 2020–December 2020. Clinical Infectious Diseases 2021 ciab626, https://doi.org/10.1093/cid/ciab626

**Appendix Text 2:** India and non-high income countries and excess death issues

Indeed, IFR estimates in India need to be seen with extra caution. A study in Madurai in south India found an IFR of 0.043% among people >15 years old, but estimated that given the age distribution the IFR should have been 9 times larger to match other countries (1). This could reflect under-reporting of deaths, lower rates of age-adjusted death risk in India, or both (2). For example, compared with USA and European countries, India has much lower proportions of obesity, diabetes, smoking, heart disease, medically maintained patients with terminal cancer, and medically immunosuppressed patients. These are all strong risk factors for fatality from COVID-19. Also of note, excess deaths in India in 2020-2021 may be much higher than the reported COVID-19 toll (3). However, excess deaths in a single year are notoriously difficult to calculate (especially in a country with suboptimal death registration) and reflect the composite of direct deaths due to COVID-19, indirect effects of the pandemic, direct and indirect effects of the measures taken and multiple other year- and country-specific causes (4, 5). Excess deaths should not be used to calculate IFR.

Of note, our estimates of IFRs for non-elderly young strata in India are not very dissimilar to those of high-income countries, while the divergence is much stronger in the elderly population. This may be further explained by the fact that among people >70 years old, the proportion of those who are >85 years old is only 9.5% in India, while it is much higher in high income countries (e.g. Spain 23.7%, Italy 21.1%). Moreover, the difference between India and high-income countries in prevalence of major comorbidities such as obesity that increase the risk of COVID-19 mortality is most prominent in the most elderly; in younger generations in India, the influence of western lifestyle is becoming more pervasive (6, 7). Therefore, the IFR in people >70 years old in India may well be extremely lower than the respective figure in high-income countries. The same may apply also to other middle-income countries and also low-income countries.

1. Laxminarayan R, B CM, G VT, Arjun Kumar KV, Wahl B, Lewnard JA. SARS-CoV-2 infection and mortality during the first epidemic wave in Madurai, south India: a prospective, active surveillance study. Lancet Infect Dis. 2021;21(12):1665-76.

2. Li Y, Nair H. How reliable are COVID-19 burden estimates for India? Lancet Infect Dis. 2021;21(12):1615-7.

3. Anand A, Sandefur J, Subramanian A. Three new estimates of India’s all-cause excess mortality during the COVID-19 pandemic. Accessed in: <https://www.cgdev.org/publication/three-new-estimates-indias-all-cause-excess-mortality-during-covid-19-pandemic>. Last accessed, December 12, 2021: Center for Global Development, Working paper; 2021.

4. Kiang MV, Irizarry RA, Buckee CO, Balsari S. Every Body Counts: Measuring Mortality From the COVID-19 Pandemic. Annals of internal medicine. 2020;173(12):1004-7.

5. Ioannidis JPA. Over- and under-estimation of COVID-19 deaths. European journal of epidemiology. 2021;36(6):581-8.

6. Pradeepa R, Anjana RM, Joshi SR, Bhansali A, Deepa M, Joshi PP, et al. Prevalence of generalized & abdominal obesity in urban & rural India--the ICMR-INDIAB Study (Phase-I) [ICMR- NDIAB-3]. Indian J Med Res. 2015;142(2):139-50.

7. Luhar S, Timæus IM, Jones R, Cunningham S, Patel SA, Kinra S, et al. Forecasting the prevalence of overweight and obesity in India to 2040. PloS one. 2020;15(2):e0229438.
